# Supplementary material for: Photonic spin-Hall effect in chiral plasmonic assemblies
Source: Nat Commun. 2026 Feb 26;17:3246. doi: 10.1038/s41467-026-70039-5 (PMC13061901; doi:10.1038/s41467-026-70039-5)
Supplement: Supplementary file 1 — Supplementary Information [file 41467_2026_70039_MOESM1_ESM.pdf]

Supplementary information

Photonic spin-Hall effect in chiral plasmonic assemblies

Authors: Yilin Chen[1,2,†], Yang Chen[1,†], Yini Fang[1], Ruoqi Ai[1,3], Ximin Cui[3], Xiaolu Zhuo[2]* & Jianfang Wang[1]*

Affiliations:

[1]Department of Physics, The Chinese University of Hong Kong, Shatin, Hong Kong SAR 999077, China

[2]School of Science and Engineering, The Chinese University of Hong Kong, Shenzhen, Guangdong 518172, China

[3]College of Electronics and Information Engineering, Shenzhen University, Shenzhen, Guangdong 518060, China

[†]These authors contributed equally to this work.

*Corresponding author. Email: zhuoxiaolu@cuhk.edu.cn (X.L.Z.); jfwang@phy.cuhk.edu.hk (J.F.W.)

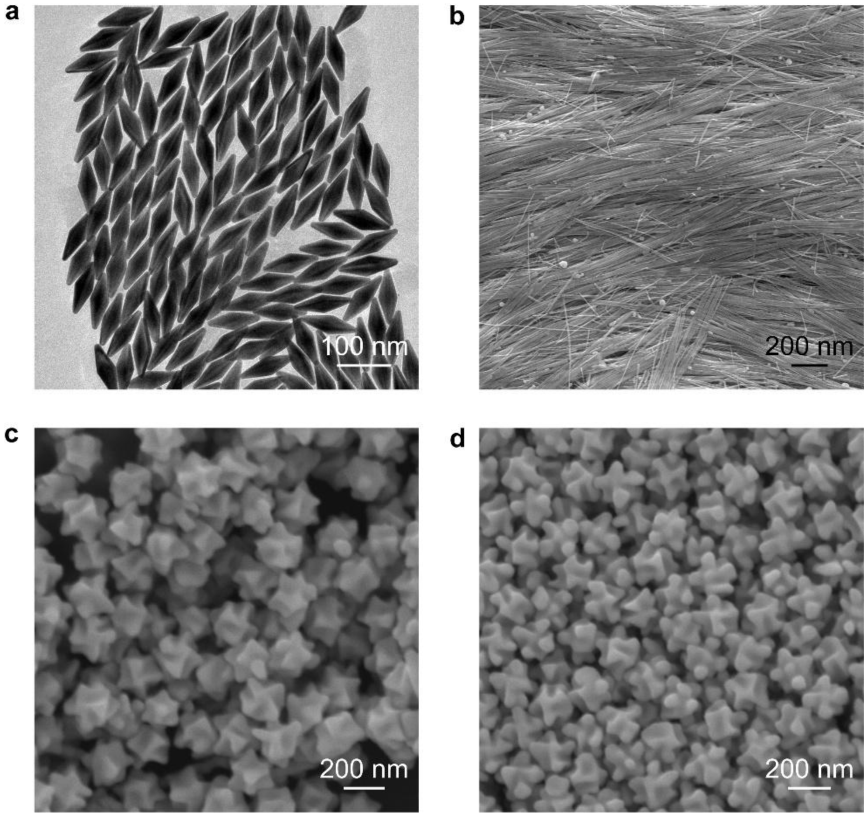

Supplementary Fig. 1 | Au nanobipyramids, Ag nanowires, and chiral Au nanocubes. a Transmission electron microscopy image of the Au nanobipyramids. **b** Scanning electron microscopy (SEM) image of the Ag nanowires. **c**, **d** SEM images of the D-handed (**c**) and L-handed chiral Au nanocubes (**d**).

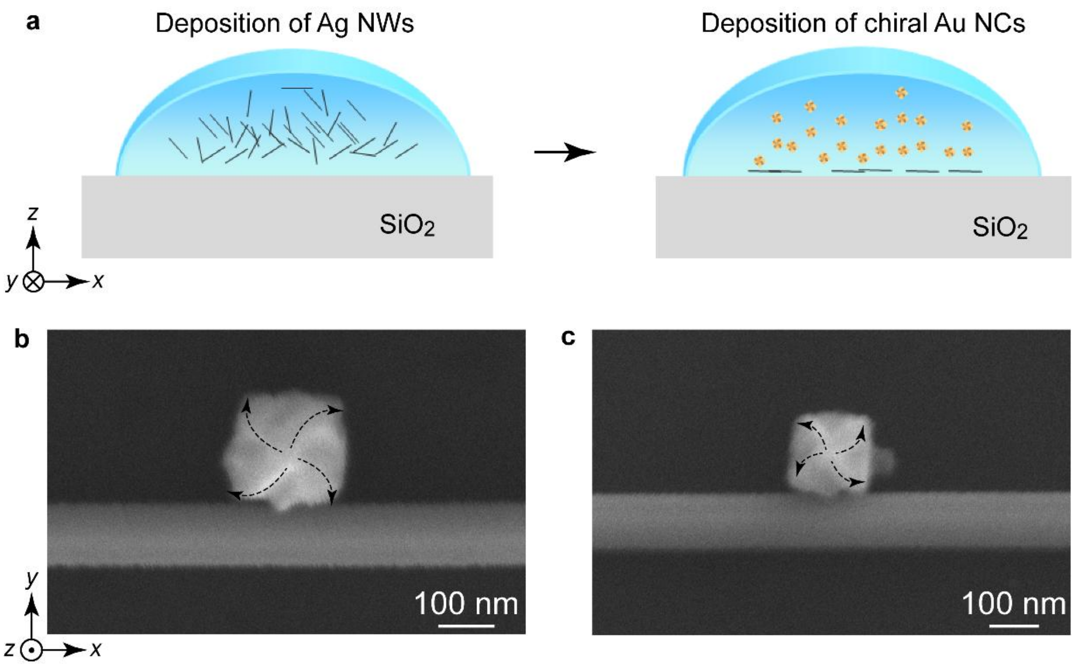

Supplementary Fig. 2 | (Chiral Au nanocube)–(Ag nanowire) structures. a Schematic of the assembling process of the chiral Au nanocube–nanowire (NC–NW) structures. **b**, **c** Scanning electron microscopy images of the (D-handed chiral Au NC)–NW (**b**) and (L-handed chiral Au NC)–NW structures (**c**). The chiral Au NC sample exhibits a distinct twisted-arm structure with fourfold rotational symmetry. The curvature direction of a half-ring along the arm, clockwise or counterclockwise, can be assigned as D- or L-handedness, respectively.

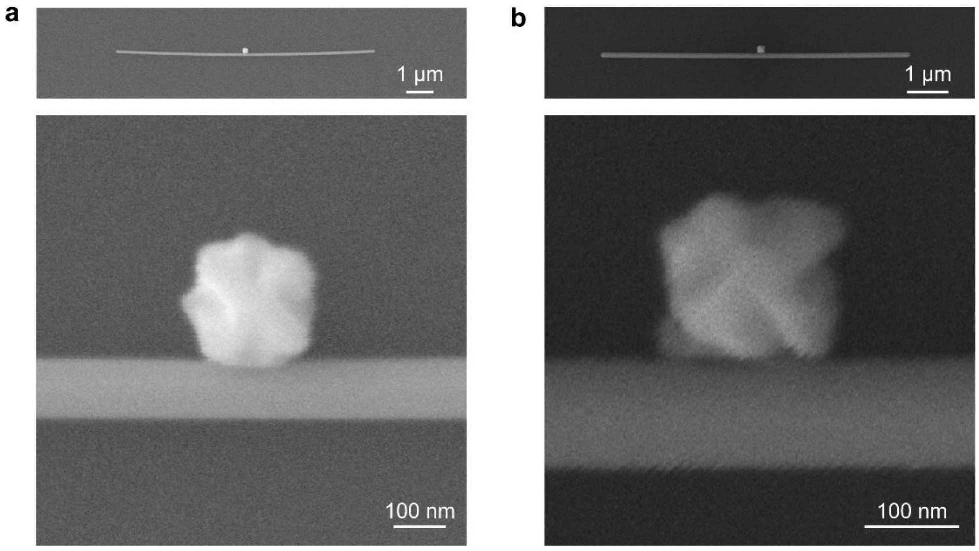

Supplementary Fig. 3 | Scanning electron microscopy images of the (chiral Au nanocube)–(Ag nanowire) structures. a (D-handed chiral Au nanocube)–(Ag nanowire) structure. **b** (L-handed chiral Au nanocube)–(Ag nanowire) structure.

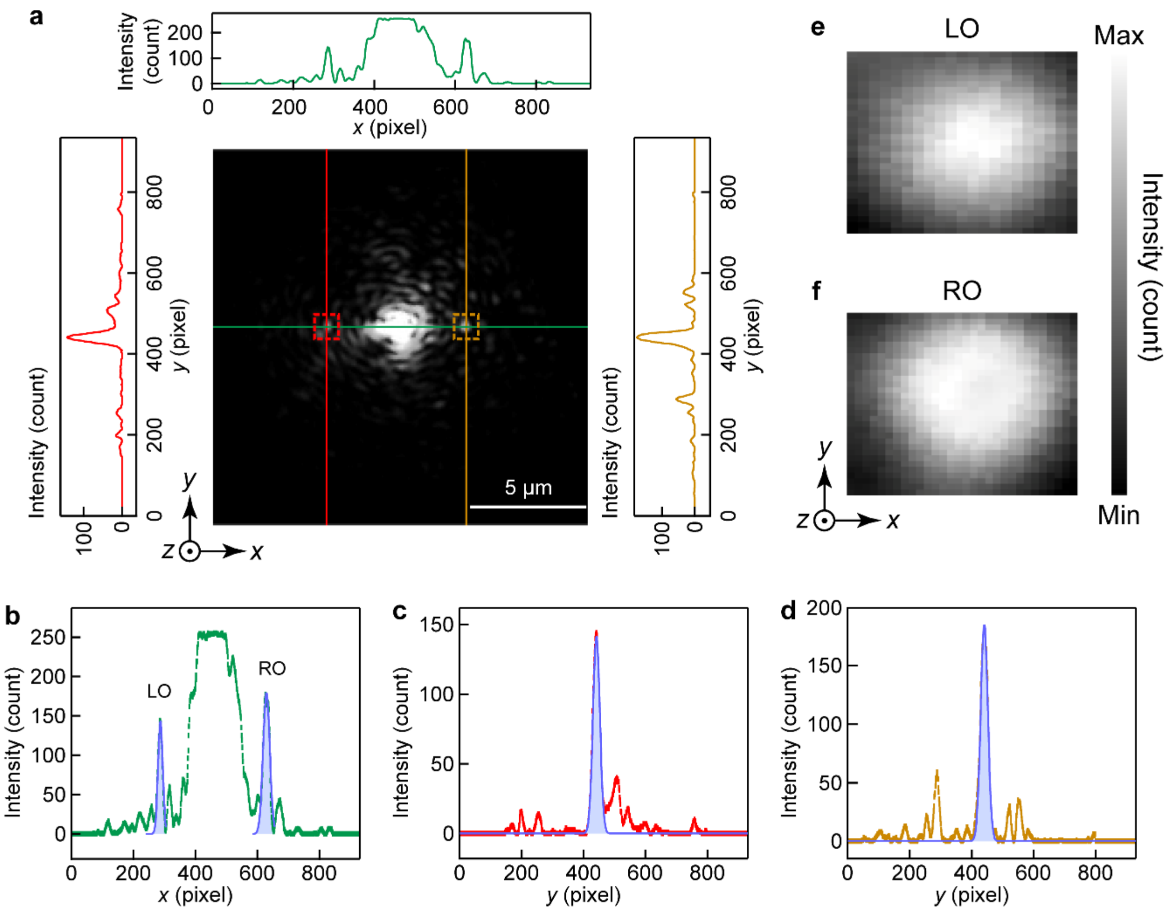

Supplementary Fig. 4 | Determination of the left and right output intensities. a Captured charge-coupled device (CCD) image for left-handed circularly polarized (LCP) laser illumination at the D-handed chiral Au nanocube in an ideal chiral waveguide and the intensity profiles along the green, red, and yellow lines. The regions of the left and right output (LO and RO) were first approximately selected in the CCD image as shown in the red and yellow boxes, respectively. The maximal intensities in the selected regions determine their central positions. A green line was drawn along the x-direction passing through the central position of the LO and RO. Red and yellow lines were drawn along the y-direction passing through the central positions of the LO and RO, respectively. **b–d** Gaussian fitting of the intensity profiles of the green (**b**), red (**c**), and yellow lines (**d**). The widths in the x and y directions of the LO and RO were determined by measuring the widths at $1/e$ of the maximal intensities in the profiles, respectively. The regions of the LO and RO were therefore determined by their x-widths and

y-widths. **e**, **f** Intensity distributions in the designated regions of the LO (**e**) and RO (**f**), with brighter areas representing higher intensities and darker areas representing lower intensities. The intensity counts in the cut CCD images of the LO and RO were summed to calculate the intensities of the LO and RO, respectively.

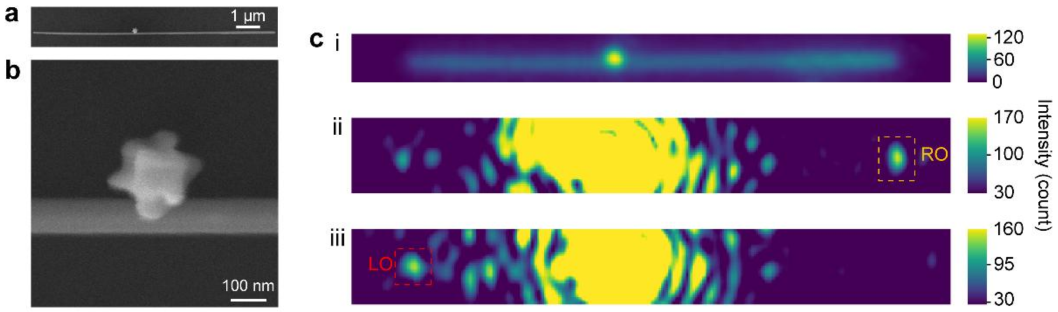

Supplementary Fig. 5 | Spin-direction locking behavior for the chiral Au nanocube. a, **b** Scanning electron microscopy images of a D-handed chiral Au nanocube–nanowire (NC–NW) structure. The chiral Au NC is located to the left of the central position of the Ag NW and subjected to spatial rotation relative to the ideal chiral waveguide. **c** Pseudocolor dark-field microscopy image of the (D-handed chiral Au NC)–NW structure (i) and pseudocolor images for the illumination of left- (ii) and right-handed circularly polarized (LCP and RCP) laser light (iii). The LCP and RCP laser light were directionally coupled toward the right output (RO, yellow box) and left output (LO, red box) of the NW, respectively.

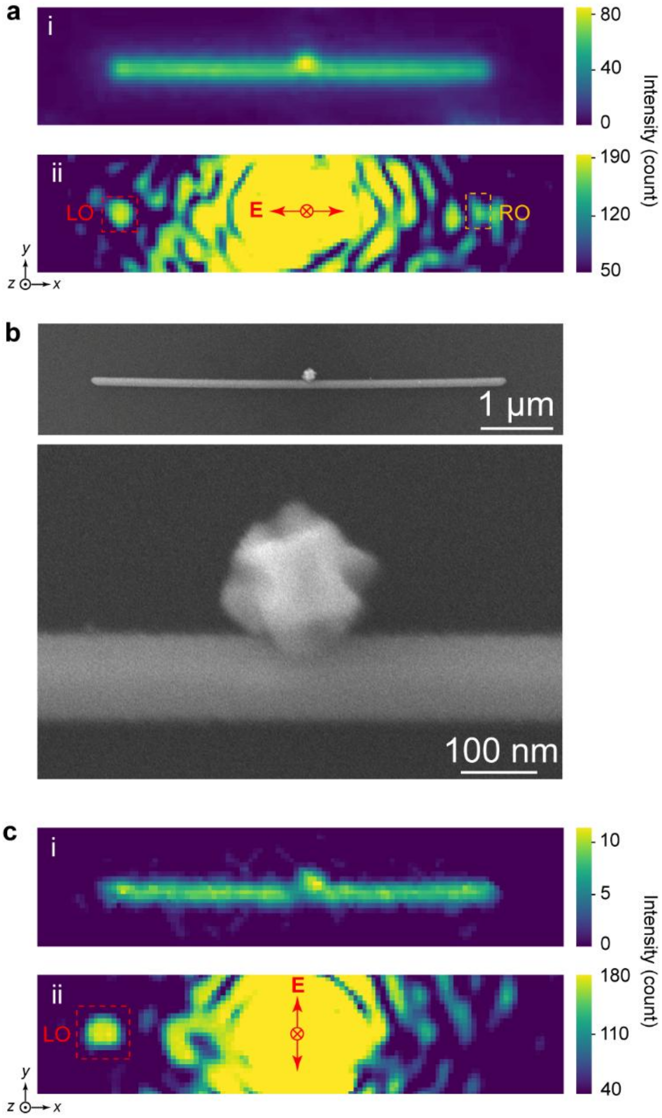

Supplementary Fig. 6 | Directional surface plasmon propagation under the excitation of linearly polarized light. a Pseudocolor dark-field microscopy image of a L-handed chiral Au nanocube–nanowire (NC–NW) structure (i) and pseudocolor image for the linearly polarized laser illumination of the L-handed chiral Au NC (ii). The linearly polarized laser light was directionally coupled toward the left output (LO, red box). The polarization of the excitation light is along the *x* axis. **b** Scanning electron microscopy images of a different (L-handed chiral Au NC)–(Ag NW) structure. **c** Pseudocolor dark-field microscopy image of the (L-handed chiral Au NC)–NW structure (i) and pseudocolor image for the linearly polarized laser illumination of the L-handed chiral Au NC (ii). The linearly polarized laser light was directionally coupled toward the LO (red box). The polarization of the excitation light is along

the y axis.

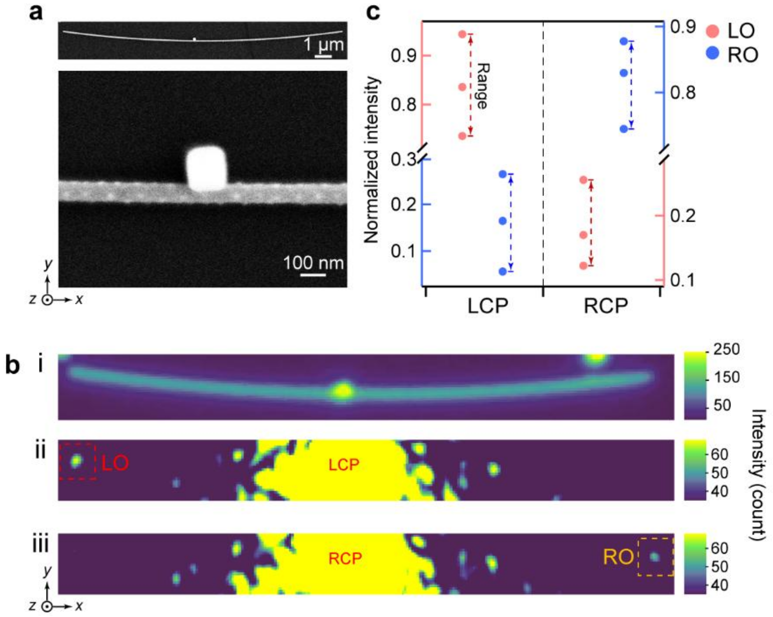

Supplementary Fig. 7 | Reversed photonic spin-Hall effect in the (achiral Au nanocube)–(Ag nanowire) structures. a Scanning electron microscopy images of an achiral nanocube–nanowire (NC–NW) structure. The Au NC is located at the central position of the Ag NW. The edge length of the Au NC is ~130 nm. **b** Pseudocolor dark-field microscopy image of the (Au NC)–NW structure (i) and pseudocolor images for the illumination of left- (ii) and right-handed circularly polarized (LCP and RCP) laser light (iii). The LCP and RCP laser light are directionally coupled toward the left output (LO, red box) and right output (RO, yellow box) of the NW, respectively. **c** Normalized intensities of the LO and RO of the surface plasmon polariton (SPP) propagation in the (Au NC)–NW structures under the excitation of LCP and RCP light. The data were collected and averaged from 3 (Au NC)–NW structures, respectively. The dashed arrows in (**c**) represent the range of measured normalized intensities. Normal photonic spin-Hall effect in Fig. 1c is defined as the scenario in which under the excitation of circularly polarized light (CPL) along the –*z* direction, LCP- and RCP-excited SPPs propagate toward the RO and LO of the NW, respectively. In contrast, the reversed spin-Hall effect in (**b**)

describes the flipped case where under the same incident CPL along the –*z* direction, LCP- and RCP-excited SPPs propagate toward the LO and RO, respectively.

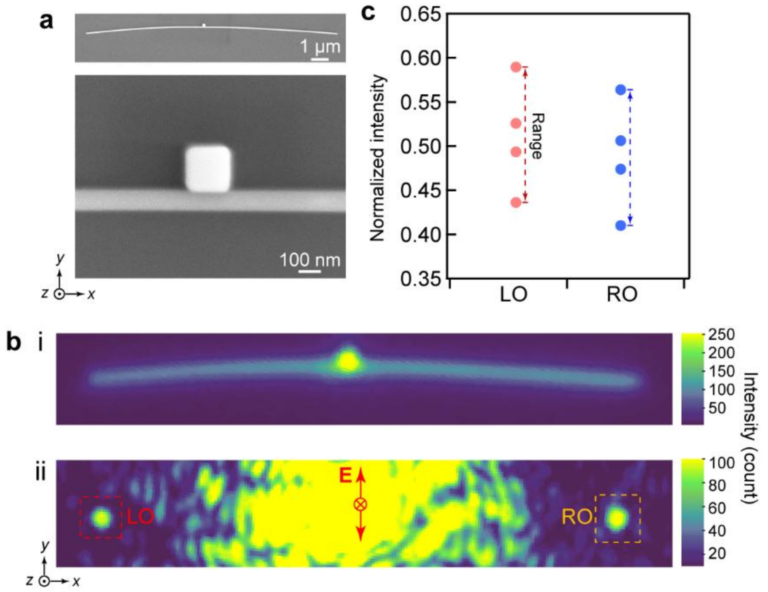

Supplementary Fig. 8 | Surface plasmon propagation in the (achiral Au nanocube)–(Ag nanowire) structures under the excitation of linearly polarized light. a Scanning electron microscopy images of an achiral nanocube–nanowire (NC–NW) structure. The Au NC is located at the central position of the Ag NW. **b** Pseudocolor dark-field microscopy image of the (Au NC)–NW structure (i) and pseudocolor image for the illumination of linearly polarized laser light (ii). The output intensity difference between the right output (RO, yellow box) and left output (LO, red box) of the NW is insignificant. **c** Normalized intensities of the LO and RO of the surface plasmon polariton propagation in the (Au NC)–NW structures under the excitation of linearly polarized laser light. The data were collected and averaged from 4 (Au NC)–NW structures. The dashed arrows in (**c**) represent the range of measured normalized intensities.

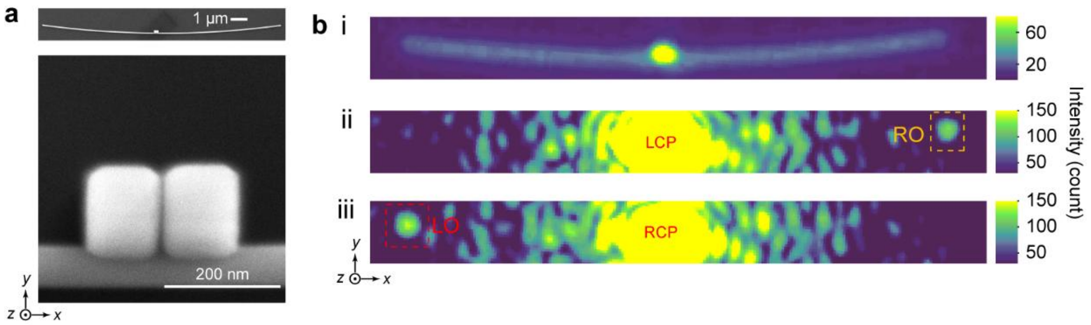

Supplementary Fig. 9 | Photonic spin-Hall effect in a (Au nanocube dimer)–(Ag nanowire) structure. a Scanning electron microscopy images of a hybrid structure of a Au nanocube (NC) dimer and a nanowire (NW). The Au NC dimer is located at the central position of the Ag NW. **b** Pseudocolor dark-field microscopy image of the (Au NC dimer)–NW structure (i) and pseudocolor images for the illumination of left- (ii) and right-handed circularly polarized (LCP and RCP) laser light (iii). The LCP and RCP laser light were directionally coupled toward the right output (RO, yellow box) and left output (LO, red box) of the NW, respectively.

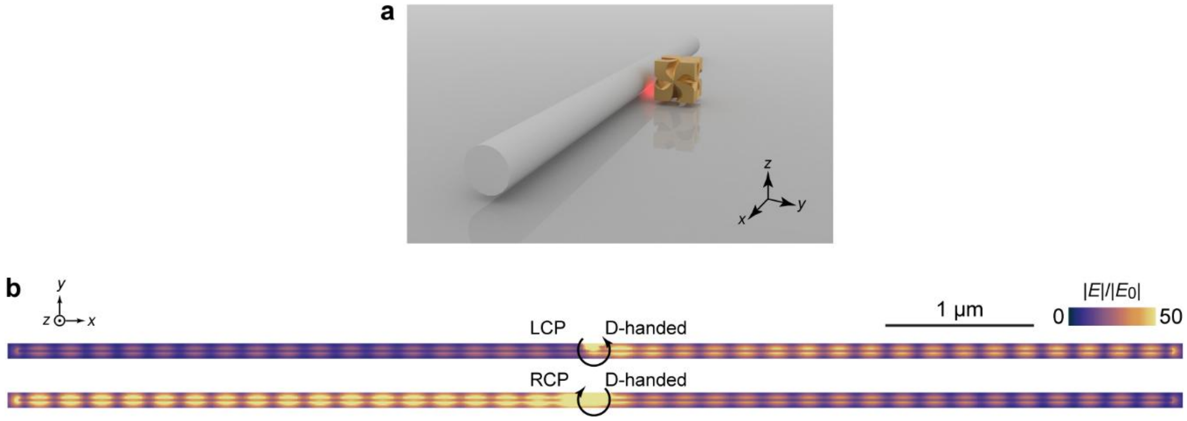

Supplementary Fig. 10 | Simulations of the electric field of the (D-handed chiral Au nanocube)–(Ag nanowire) structure. a Schematic of a dipole source located between a Ag nanowire (NW) and a D-handed chiral Au nanocube (NC). **b** Distributions of the electric field enhancement in an *x*-*y* plane monitor located at the bottom surface of the Ag NW under the excitation of left- and right-handed circularly polarized (LCP and RCP) dipoles, respectively. Surface plasmon polaritons (SPPs) propagate along the +*x* direction under the excitation of the LCP dipole, whereas the RCP excitation launches SPPs along the −*x* direction.

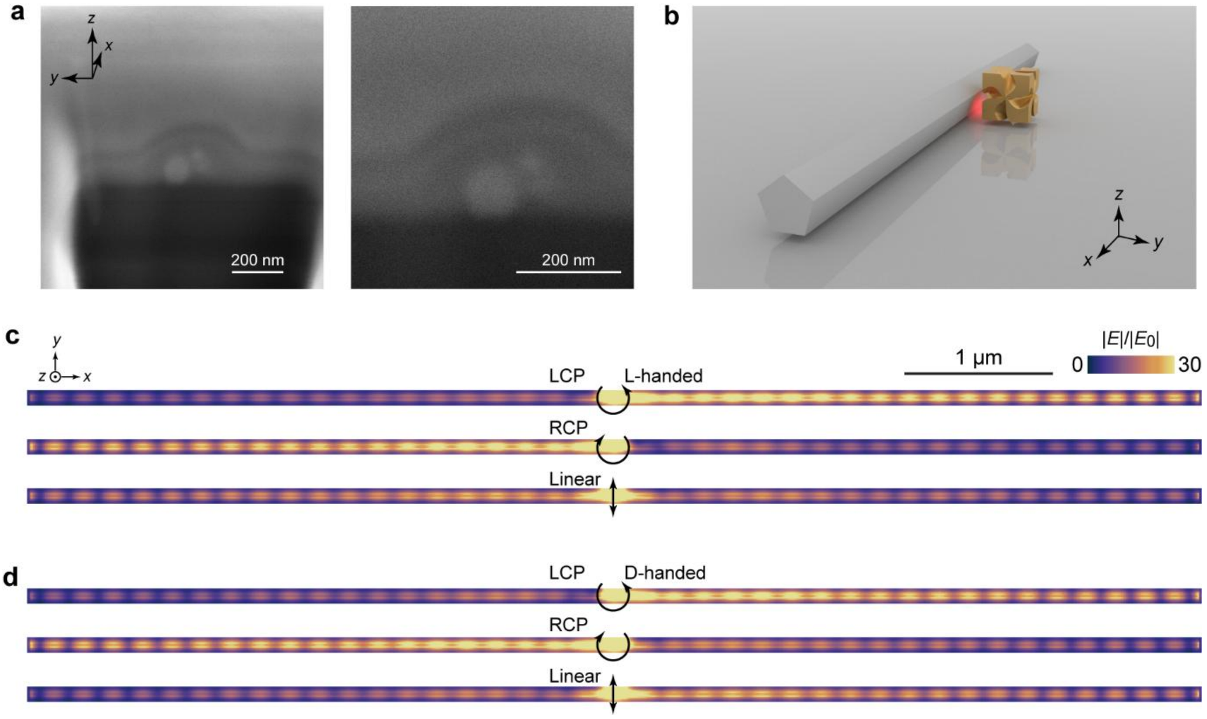

Supplementary Fig. 11 | Simulations of the electric field of the (chiral Au nanocube)–(faceted pentagonal Ag nanowire) structure. **a** Cross-sectional morphology of a Ag nanowire (NW) characterized by focused ion beam (FIB) milling and tilted scanning electron microscopy imaging. The FIB milling and imaging were carried out on a dual-beam FIB system (Scios 2 DualBeam by Thermo Scientific). **b** Schematic of a dipole source located between a faceted pentagonal Ag NW and a chiral Au nanocube (NC). **c** Distributions of the electric field enhancement in an *x*-*y* plane monitor located at the bottom surface of the Ag NW in the (L-handed chiral Au NC)−(faceted pentagonal Ag NW) structure under the excitation of circularly and linearly polarized dipole sources. The polarization direction of the linearly polarized dipole is perpendicular to the longitudinal axis of the Ag NW. Surface plasmon polaritons (SPPs) propagate along the +*x* direction for the left-handed circularly polarized (LCP) dipole, whereas SPPs propagate along the –*x* direction for the right-handed circularly polarized (RCP) and linearly polarized dipoles. **d** Distributions of the electric field enhancement in an *x*-*y* plane monitor located at the bottom surface of the Ag NW in the (D-handed chiral Au NC)−(faceted pentagonal Ag NW) structure under the excitation of circularly and linearly polarized dipole

sources. SPPs propagate along the $+x$ direction for the LCP and linearly polarized dipoles, whereas SPPs propagate along the $-x$ direction for the RCP dipole.

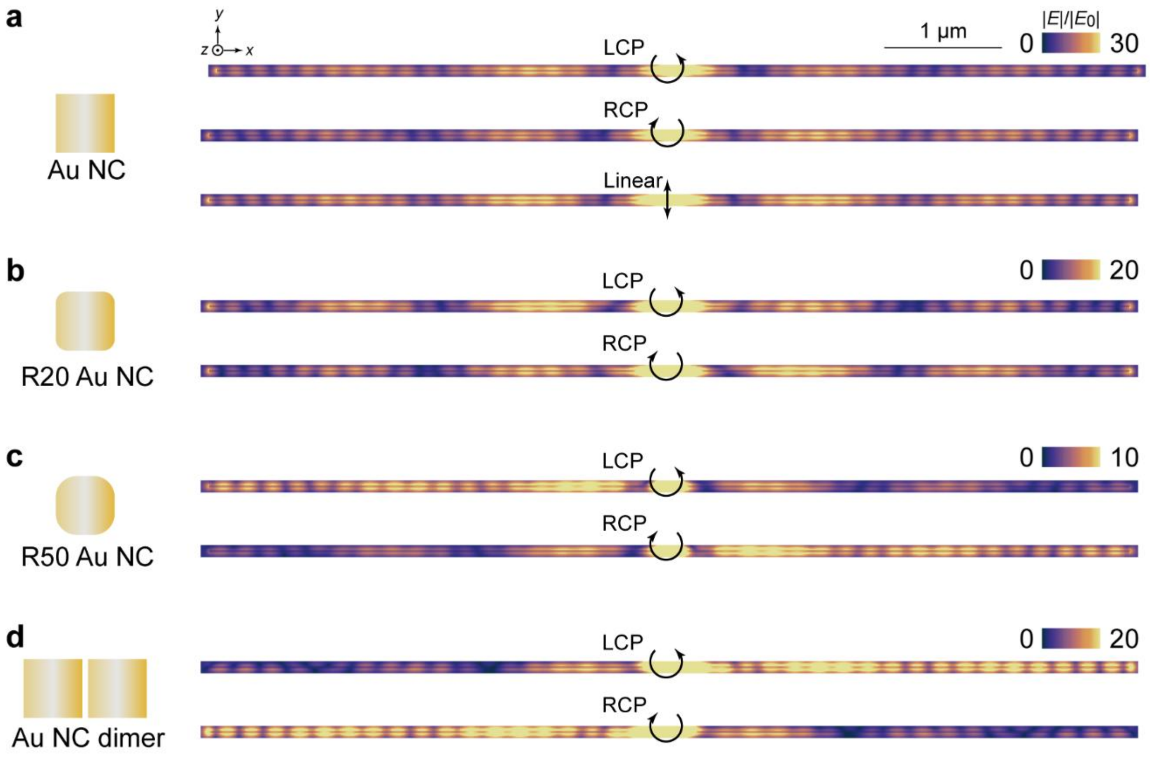

Supplementary Fig. 12 | Simulations of the electric field of the (Au nanocube)–(Ag nanowire) structures. a Distributions of the electric field enhancement in an *x*-*y* plane monitor located at the bottom surface of the Ag nanowire (NW) in the nanocube–nanowire (NC–NW) structure under the excitation of circularly and linearly polarized dipole sources. For circularly polarized dipoles, surface plasmon polaritons (SPPs) propagated along the –*x* direction for the left-handed circularly polarized (LCP) dipole, whereas SPPs propagated along the +*x* direction for the right-handed circularly polarized (RCP) dipole. For linearly polarized dipoles, no directional propagation was observed in the achiral structures. **b**, **c** Reversed photonic spin-Hall effect for the (Au NC with rounded corners)–(Ag NW) structures under the excitation of circularly polarized dipole sources. The corner-rounded Au NCs with fillet radii of 20 nm and 50 nm are denoted as R20 (**b**) and R50 Au NC (**c**), respectively. The sign of the directionality remained unchanged relative to (**a**), but the magnitude of the directionality increased as the corners of the attached NC became more rounded. **d** Normal spin-Hall effect in the (Au NC

dimer)–(Ag NW) structure under the excitation of circularly polarized dipole sources. LCP and RCP excitations were directed toward the right and left output of the NW, respectively. The position of the *x*-*y* plane monitor is identical in (**a**–**d**). The left panel schematically shows the cross-sectional configurations of the achiral Au NCs attached to the surface of the Ag NW.

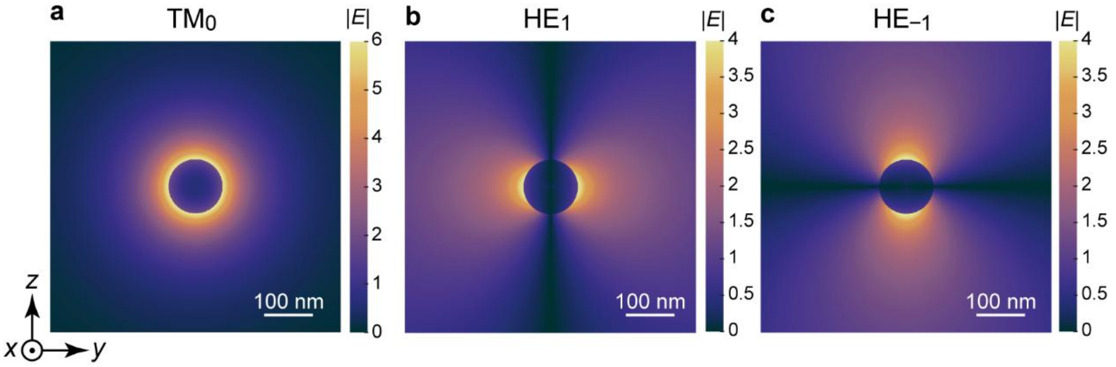

Supplementary Fig. 13 | Electric field distributions of the plasmon modes in a Ag nanowire. a TM_0 mode. The fundamental transverse magnetic mode with $m = 0$ (TM_0 mode) shows that the amplitude of the electric field varies radially. **b** HE_1 mode. **c** HE_{-1} mode. The second-order modes are the hybrid modes of HE_1 and HE_{-1} modes with $m = 1$ and $m = -1$, respectively. The HE_1 and HE_{-1} modes are doubly degenerate. The electric fields of the HE_1 and HE_{-1} modes are distributed separately on the two sides of the nanowire.

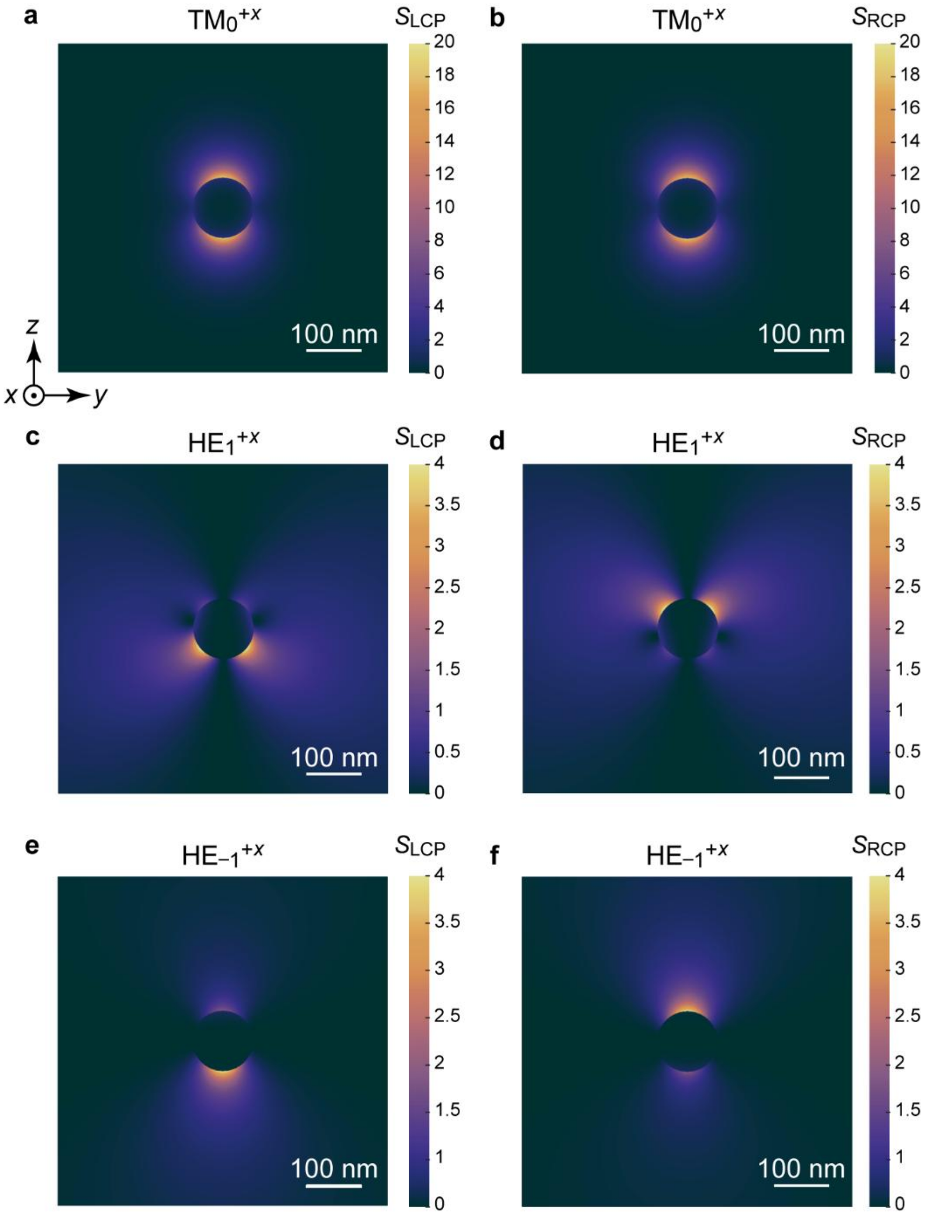

Supplementary Fig. 14 | Distributions of the overlapping intensities between the plasmon modes and incident left- and right-handed circularly polarized light propagating along the –*y* direction. a, **b** S_{LCP} (**a**) and S_{RCP} (**b**) of the TM_0^{+x} mode. Subscript LCP/RCP represents left-/right-handed circularly polarized. The S_{LCP} and S_{RCP} values of the TM_0^{+x} mode are equally distributed on the bottom surface and the upper surface of the nanowire (NW). **c**, **d** S_{LCP} (**c**) and S_{RCP} (**d**) of the HE_1^{+x} mode. The S_{LCP} value of the HE_1^{+x} mode is maximal on the bottom left and bottom right part of the NW surface. The S_{RCP} value of the HE_1^{+x} mode is maximal on the upper left and upper right part of the NW surface. **e**, **f** S_{LCP} (**e**) and S_{RCP} (**f**) of the HE_{-1}^{+x} mode.

The S_{LCP} value of the HE_{-1}^{+x} mode and the S_{RCP} value of the HE_{-1}^{+x} mode are maximal on the bottom surface and the upper surface of the NW, respectively.

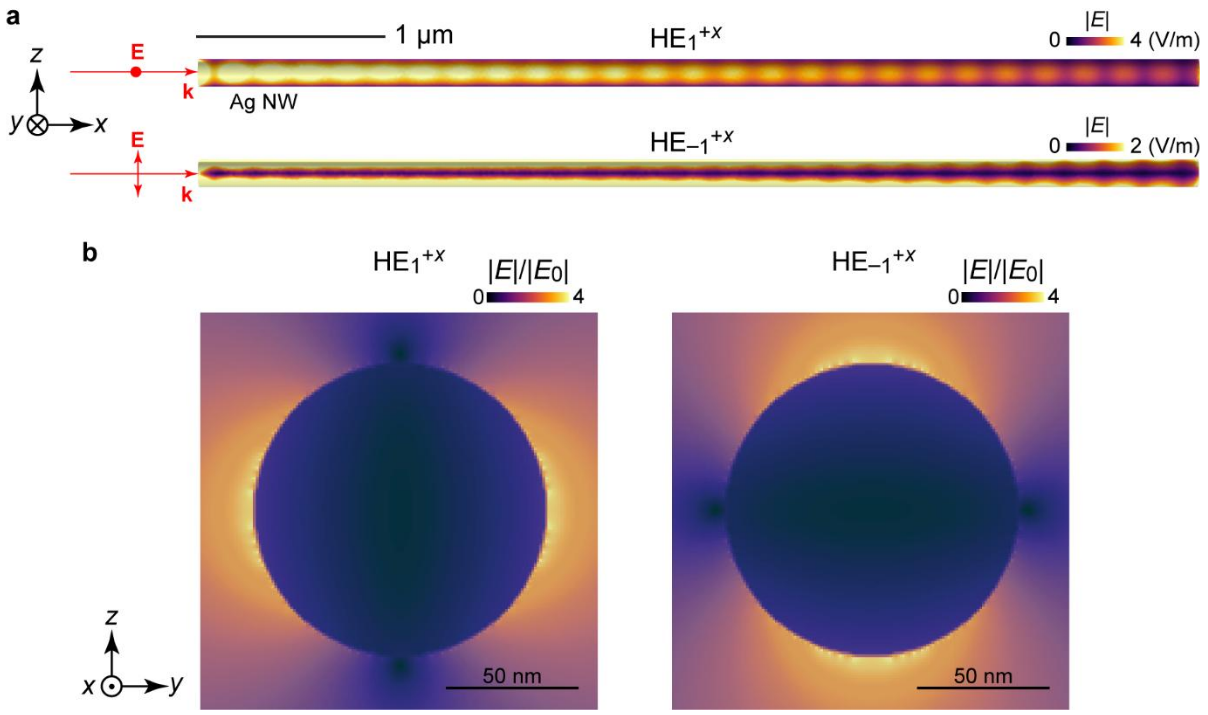

Supplementary Fig. 15 | Simulations of the electric field of the Ag nanowire under the excitation of 633 nm linearly polarized light. a COMSOL-calculated distribution of the electric field magnitude ($|E|$) on the Ag nanowire (NW) surface for the HE_1^{+x} and HE_{-1}^{+x} modes excited by linearly polarized light with *y*- and *z*-direction polarization, respectively. **b** Distribution of the electric field enhancement ($|E|/|E_0|$) in the *y*-*z* plane of the Ag NW for the HE_1^{+x} and HE_{-1}^{+x} modes under the excitation of light as shown in (**a**) calculated by the finite-difference time-domain (FDTD) method.

The electric field of the Ag NW under the excitation of 633 nm linearly polarized light was calculated by COMSOL and FDTD. In the COMSOL simulations (Supplementary Fig. 15a), the length and diameter of the Ag NW model were 8 μm and 220 nm, respectively. In the FDTD simulations (Supplementary Fig. 15b), the length and diameter of the Ag NW model were 8 μm and 110 nm, respectively. The diameters of the NW model for the COMSOL simulations were scaled to twice the actual dimensions, enabling clear resolution for the distributions of the electric field and overlapping intensities. The excited HE^{+x} mode represents surface

plasmon polaritons propagating along the +x direction. As light propagates along +x direction, the electric field magnitude decays. The plasmon oscillation direction exhibits a 90° rotation between the HE_1^{+x} and HE_{-1}^{+x} modes.

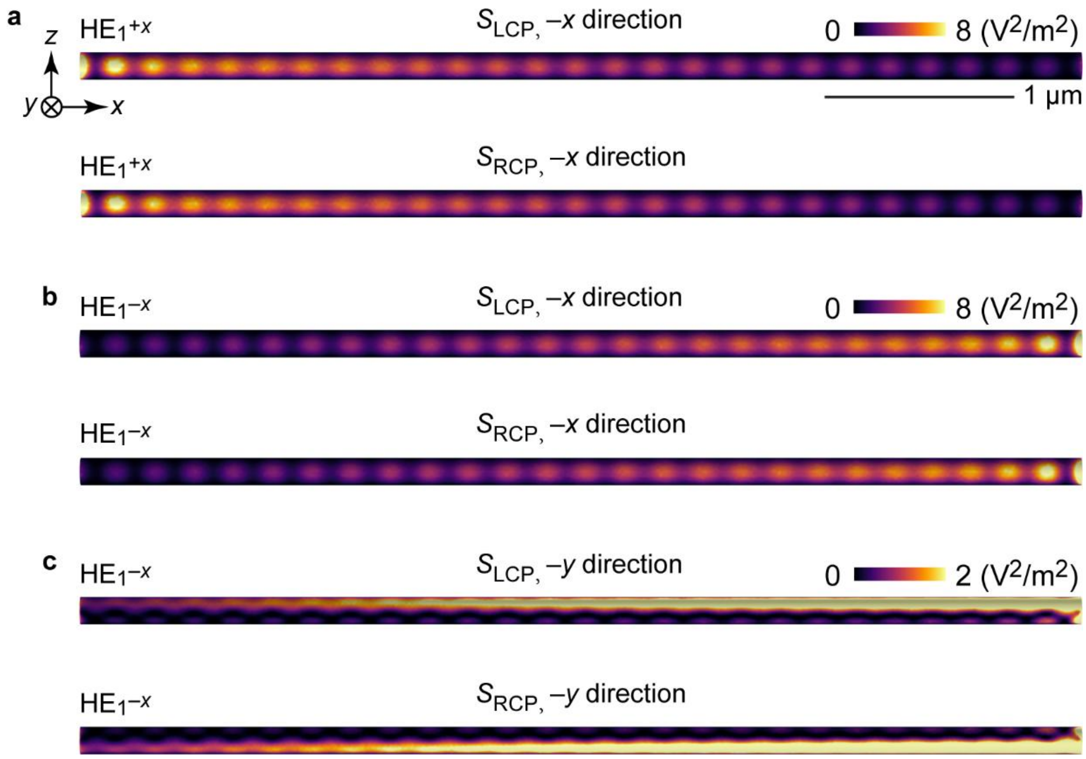

Supplementary Fig. 16 | COMSOL-calculated overlapping intensities on the Ag nanowire surface for left- and right-handed circularly polarized light and the HE_1 mode. a S_{LCP} and S_{RCP} for the overlaps between the HE_1^{+x} mode and circularly polarized light (CPL) propagating along the −*x* direction. Subscript LCP/RCP represents left-/right-handed circularly polarized. **b** S_{LCP} and S_{RCP} for the overlaps between the HE_1^{-x} mode and CPL propagating along the −*x* direction, respectively. **c** S_{LCP} and S_{RCP} for the overlaps between the HE_1^{-x} mode and CPL propagating along the −*y* direction. The distribution of the overlapping intensity between CPL and the HE_1 mode was reversed when the surface plasmon polariton propagating direction changed from +*x* (Fig. 2b) to −*x* (**c**), indicating the presence of the photonic spin-Hall effect. All the images have the same scale bar.

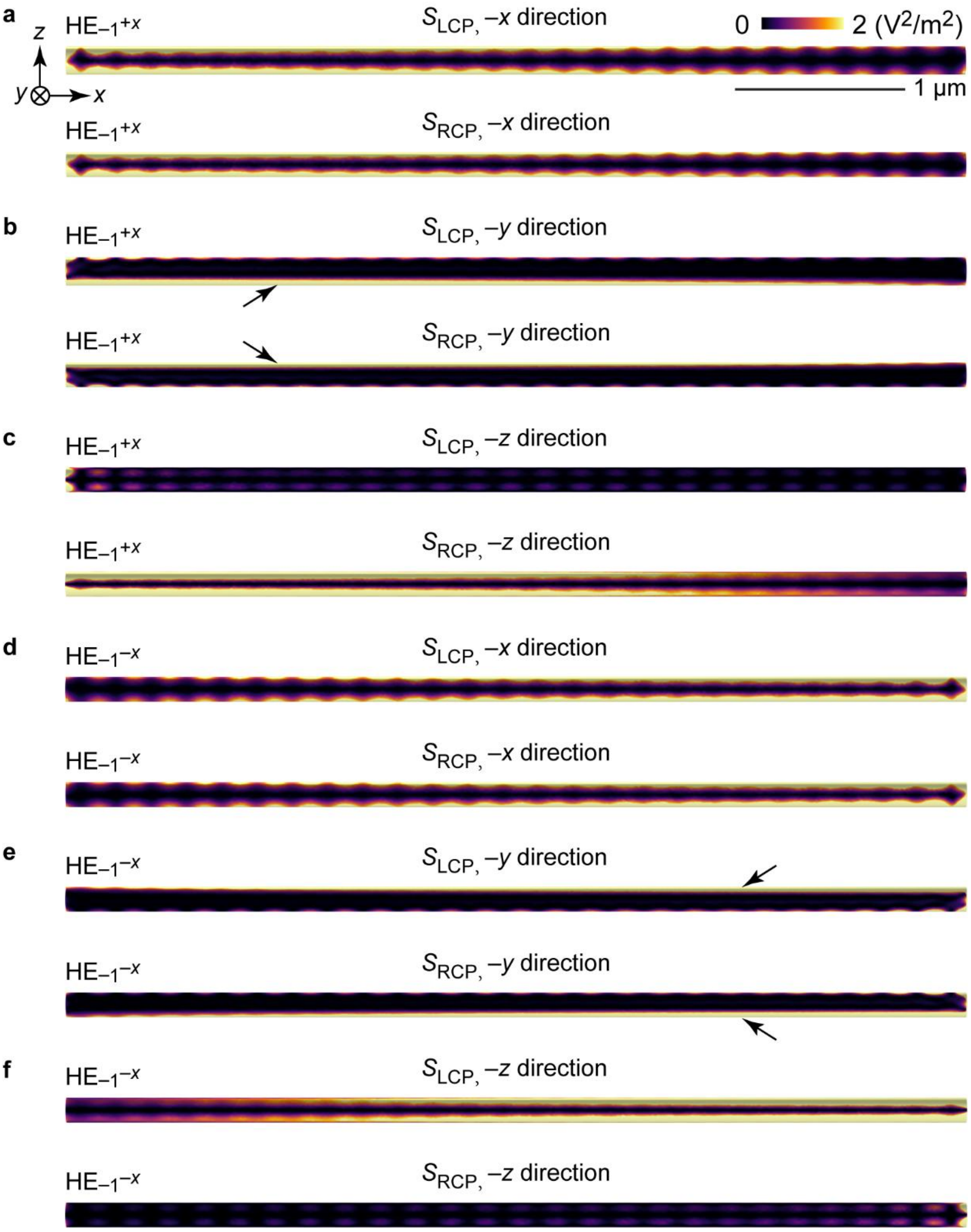

Supplementary Fig. 17 | Overlapping intensities between left- and right-handed circularly polarized light and the HE$_{-1}$ mode. a–c S_{LCP} and S_{RCP} for the overlaps between the HE$_{-1}^{+x}$ mode and circularly polarized light (CPL) propagating along the $-x$ (**a**), $-y$ (**b**), and $-z$ directions (**c**), respectively. Subscript LCP/RCP represents left-/right-handed circularly polarized. **d**–**f** S_{LCP} and S_{RCP} for the overlaps between the HE$_{-1}^{-x}$ mode and CPL propagating along the $-x$ (**d**), $-y$ (**e**), and $-z$ directions (**f**), respectively. All the images have the same scale

bar.

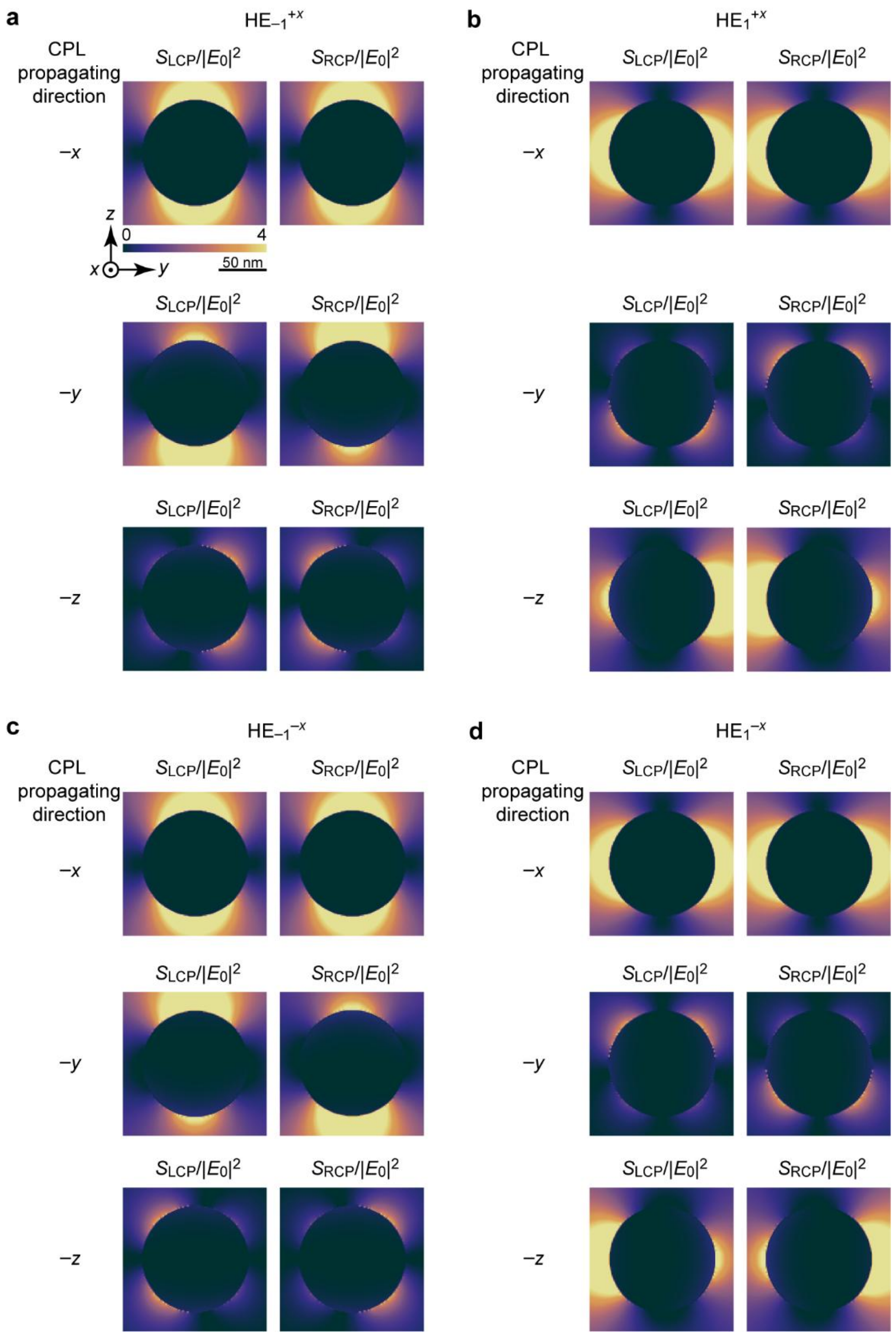

Supplementary Fig. 18 | Overlapping intensities between left- and right-handed circularly polarized light and the HE modes. a $S_{\mathrm{LCP}}/|E_0|^2$ and $S_{\mathrm{RCP}}/|E_0|^2$ for the overlaps between the $\mathrm{HE}_{-1}{}^{+x}$ mode and circularly polarized light (CPL) propagating along the different directions. Subscript LCP/RCP represents left-/right-handed circularly polarized. **b** $S_{\mathrm{LCP}}/|E_0|^2$ and $S_{\mathrm{RCP}}/|E_0|^2$ for the overlaps between the $\mathrm{HE}_1{}^{+x}$ mode and CPL propagating along different

directions. **c** $S_{LCP}/|E_0|^2$ and $S_{RCP}/|E_0|^2$ for the overlaps between the HE_{-1}^{-x} mode and CPL propagating along the different directions. **d** $S_{LCP}/|E_0|^2$ and $S_{RCP}/|E_0|^2$ for the overlaps between the HE_1^{-x} mode and CPL propagating along the different directions. All the images have the same scale bar.

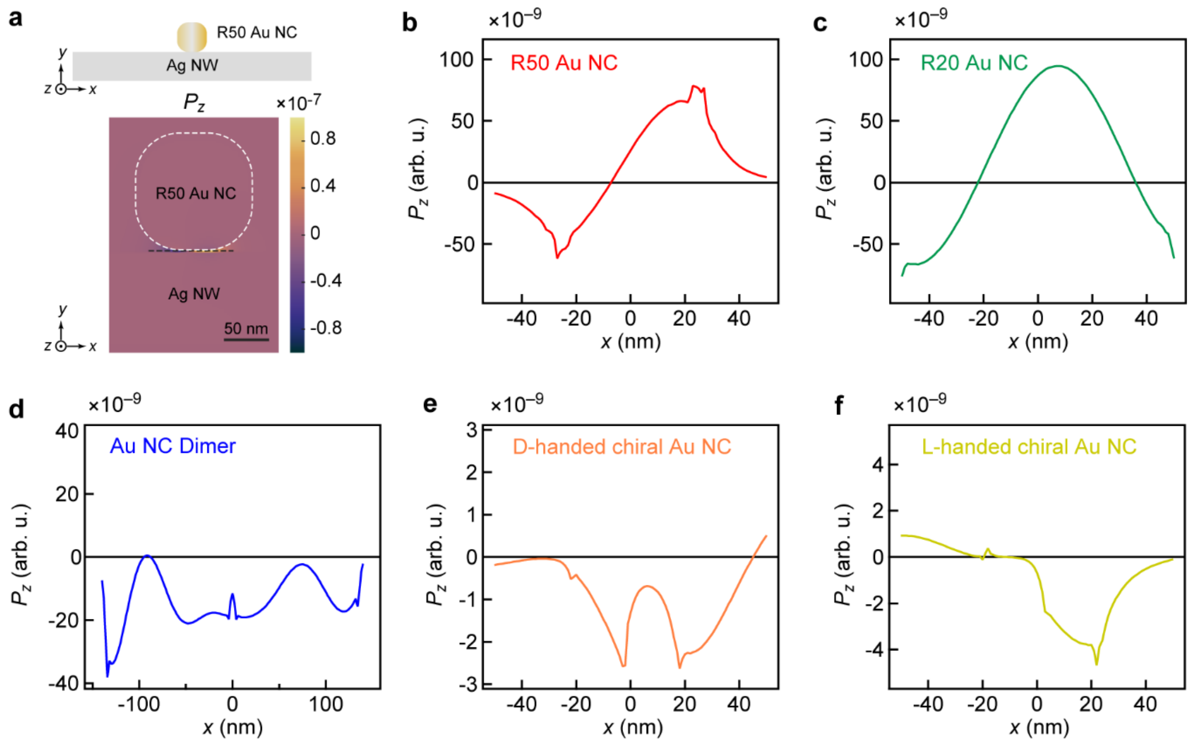

Supplementary Fig. 19 | Simulations of the *z*-component of the Poynting vector P_z under left-handed circularly polarized Gaussian beam. a Schematic of the R50 Au nanocube (NC) and Ag nanowire (NW) structure and the distribution of P_z in an *x*-*y* plane monitor. The corner-rounded Au NCs with a fillet radius of 50 nm are denoted as R50 Au NC. The red dashed line represents the position of the R50 Au NC. **b**–**f** Distributions of P_z in the line at the gap between the plasmonic nanoparticle and the Ag NW as shown in the black dashed line of (**a**). The plasmonic nanoparticles are R50 Au NC (**b**), R20 Au NC (**c**), Au NC dimer (**d**), D-handed chiral Au NC (**e**), and L-handed chiral Au NC (**f**), respectively. The directions of P_z in the (R50 Au NC)–NW and (R20 Au NC)–NW structures (**b**, **c**) are along the +*z* direction, which are opposite to those of the cases with the Au NC dimer and the chiral Au NCs (**d**–**f**).

The reversal between the normal and reversed photonic spin-Hall effects can be attributed to the sign change of P_z induced by the plasmon coupling between the nanoparticle and the NW.

For LCP light incident along the $-z$ direction, the plasmonic interaction in the gap region flips the sign of P_z, as evidenced by its distribution along a line across the nanoparticle–NW gap (Supplementary Fig. 19). P_z is negative (pointing in the $-z$ direction) for structures with an attached Au NC dimer or D-/L-handed chiral Au NC, whereas P_z is positive (pointing in the $+z$ direction) for those with the R50 or R20 Au NC. Since the opposite propagating directions of light in the coupling fields give rise to opposite SPP coupling directions, this sign reversal of P_z from negative to positive accounts for the transition from the normal to the reversed spin-Hall effect. This interpretation is consistent with the simulation results under the excitation of circularly polarized dipole sources in Supplementary Figs. 10, 12 and Fig. 2a.

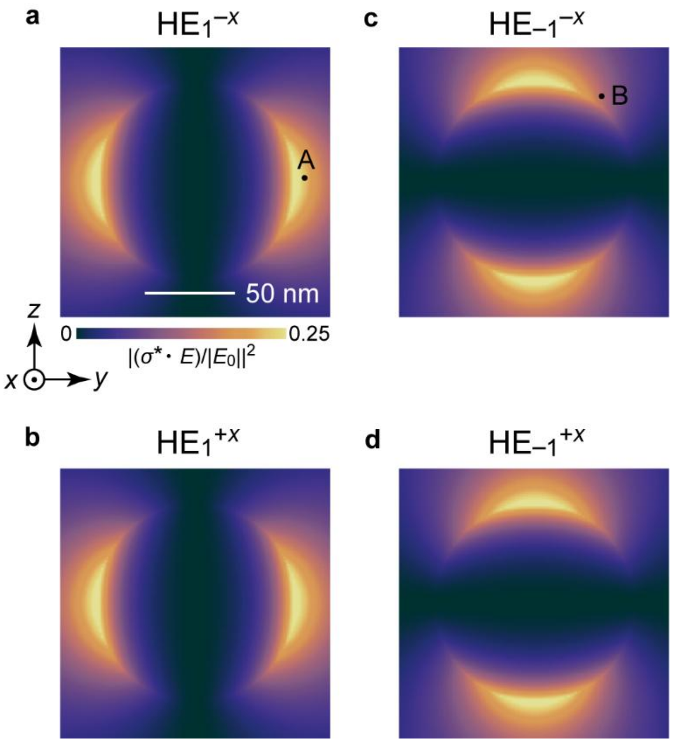

Supplementary Fig. 20 | Overlapping intensities between linearly polarized light propagating along the −*z* direction and the HE modes. a $S_\sigma/|E_0|^2$ for the overlap between the HE_1^{-x} mode and linearly polarized light with polarization ellipticity $\varepsilon = 0$. S_σ is defined as $|\sigma^* \cdot \mathbf{E}(y, z)|^2$. **b** $S_\sigma/|E_0|^2$ for the overlap between the HE_1^{+x} mode and linearly polarized light with $\varepsilon = 0$. **c** $S_\sigma/|E_0|^2$ for the overlap between the HE_{-1}^{-x} mode and linearly polarized light with $\varepsilon = 0$. **d** $S_\sigma/|E_0|^2$ for the overlap between the HE_{-1}^{+x} mode and linearly polarized light with $\varepsilon = 0$. All the images have the same scale bar. The points A and B adjacent to the +*y* side of the nanowire surface were selected to demonstrate the ε-dependence of the overlapping intensities in Fig. 2c.

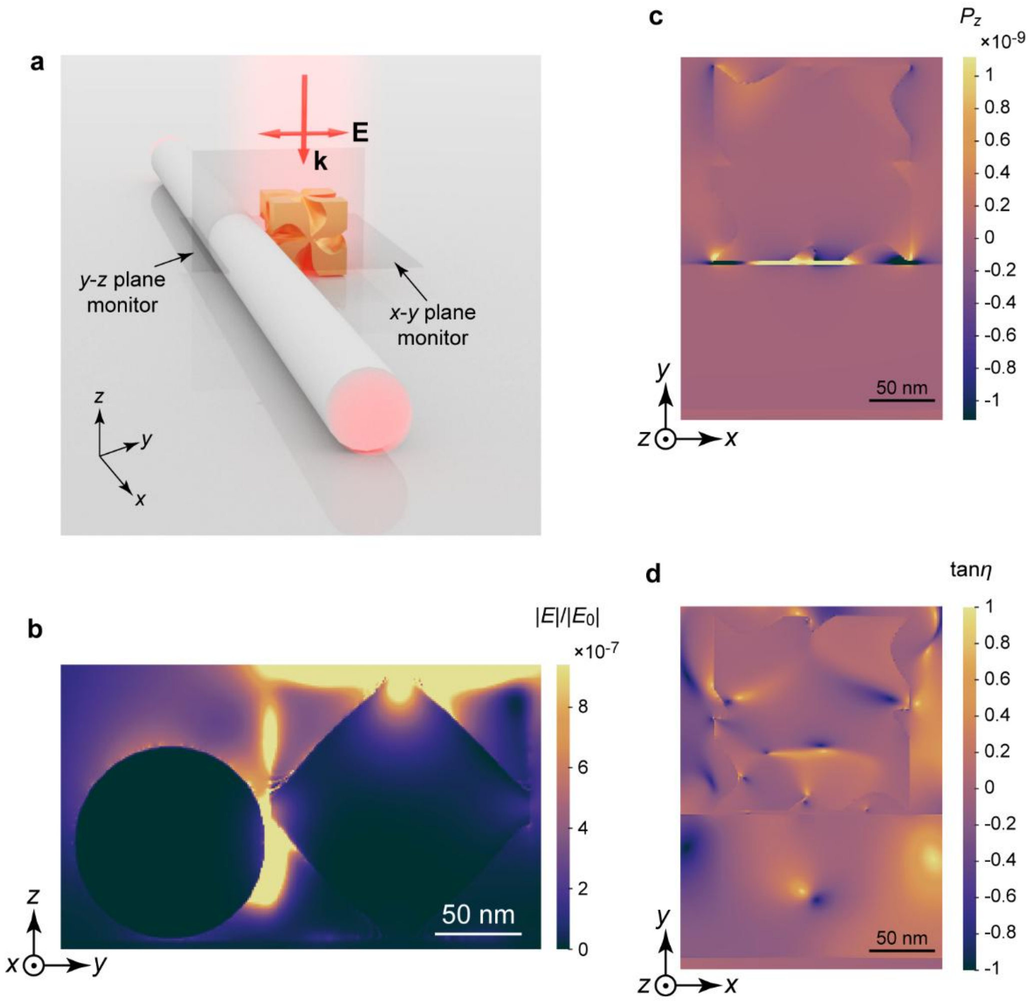

Supplementary Fig. 21 | Distribution of the strength of ellipticity of a (L-handed chiral Au nanocube)–(Ag nanowire) structure. a Schematic of the surface plasmon polariton excitation of the (L-handed chiral Au nanocube)–nanowire structure under the illumination of 633 nm linearly polarized light. The edge length of the chiral Au nanocube model is 150 nm. The grey regions represent the *x*-*y* plane and *y*-*z* plane regions of interest (ROIs) for the simulations. The ROIs pass through the center of the nanowire. **b** Distribution of the electric field in the *y*-*z* plane ROI. **c**, **d** Distributions of the *z*-component of the Poynting vector P_z (**c**) and the elliptical polarization of the electric field $\tan\eta$ (**d**) in the *x*-*y* plane ROI. $\tan\eta$ is used to characterize the elliptical polarization of the electric field. For example, the sign of $\tan\eta$ at a certain position is reversed from +1 to −1 when the circular polarization of the wavevector pointing in one direction is changed from left to right-handed circularly polarized.

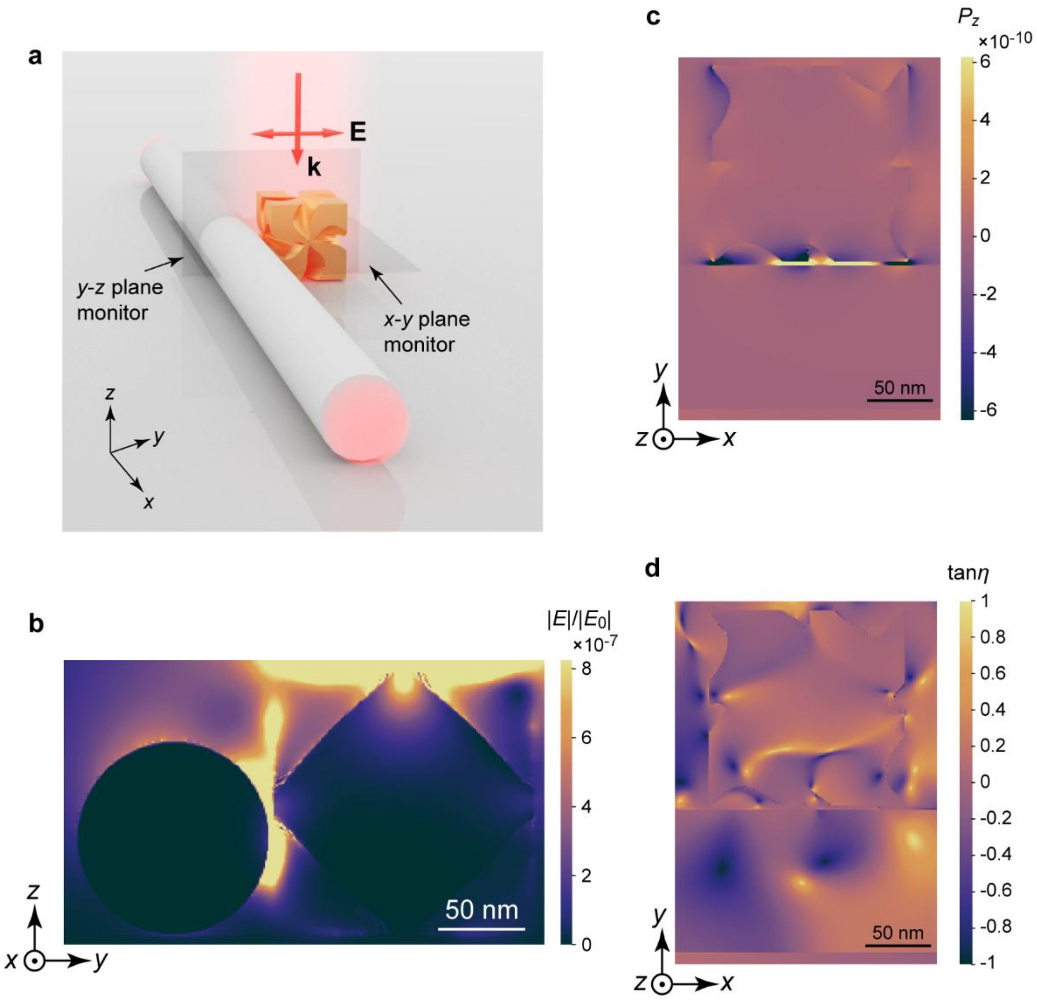

Supplementary Fig. 22 | Distribution of the strength of ellipticity of a (D-handed chiral Au nanocube)–(Ag nanowire) structure. a Schematic of the surface plasmon polariton excitation of the (D-handed chiral Au nanocube)–nanowire structure under the illumination of 633 nm linearly polarized light. The grey regions represent the *x*-*y* plane and *y*-*z* plane regions of interest (ROIs) for the simulations. **b** Distribution of the electric field in the *y*-*z* plane ROI. **c**, **d** Distributions of P_z (**c**) and tanη (**d**) in the *x*-*y* plane ROI.

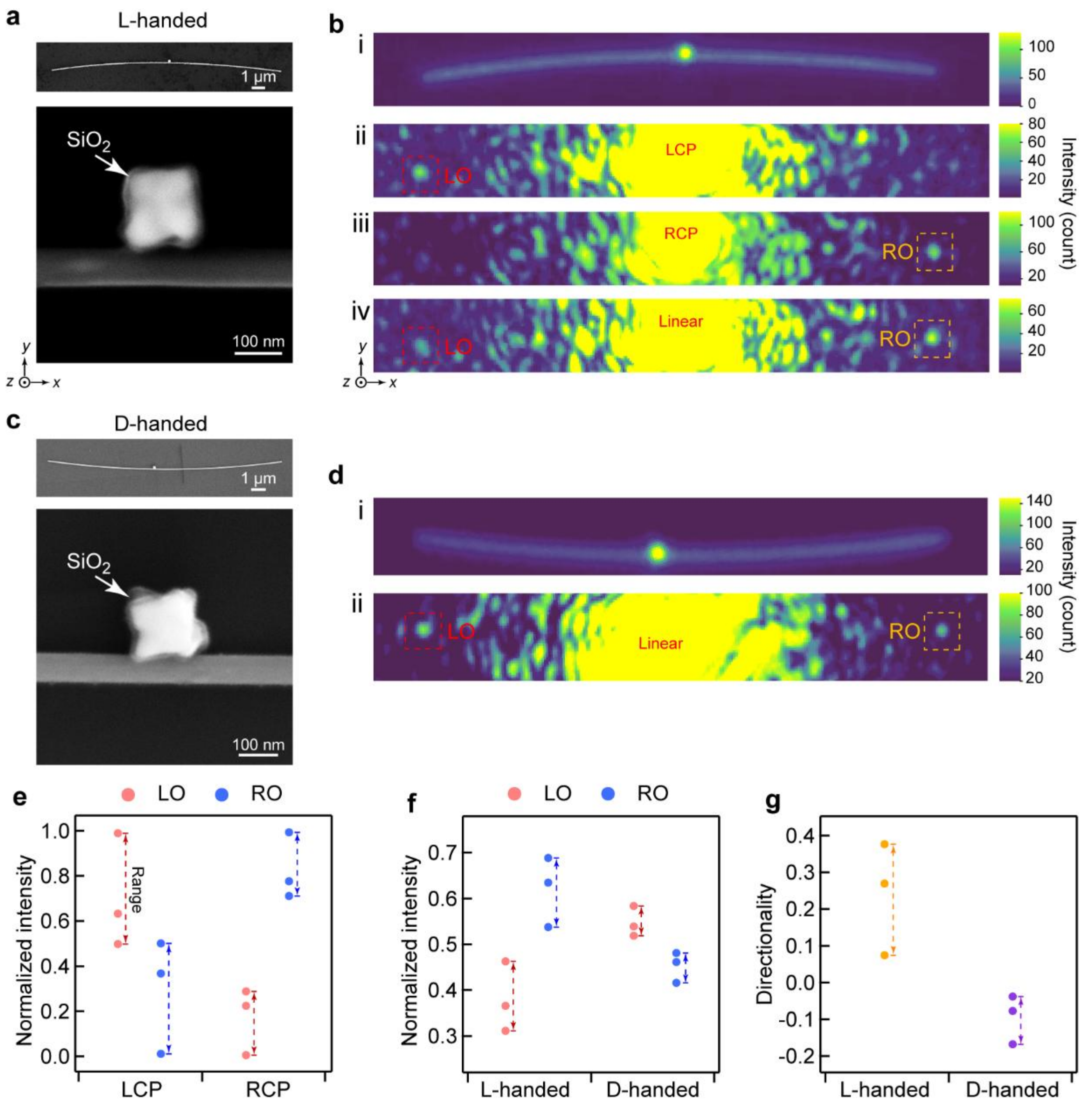

Supplementary Fig. 23 | Surface plasmon polariton propagation in the (chiral Au nanocube)@silica–(Ag nanowire) structures. a Scanning electron microscopy (SEM) images of an L-handed nanocube@silica–nanowire (NC@SiO_2–NW) structure. **b** Pseudocolor dark-field microscopy image of the (L-handed chiral Au NC)@SiO_2–NW structure (i) and pseudocolor images for the illumination of left-handed circularly polarized (LCP) (ii), right-handed circularly polarized (RCP) (iii), and linearly polarized laser light (iv). The direction of linear polarization is perpendicular to the long axis of the NW. The LCP and RCP laser light were directionally coupled toward the left output (LO, red box) and right output (RO, yellow box) of the NW, respectively. The linearly polarized laser light was directionally coupled toward the RO (yellow box) of the NW. **c** SEM images of a (D-handed chiral Au NC)@SiO_2–

NW structure. **d** Pseudocolor dark-field microscopy image of the (D-handed chiral Au NC)@SiO_2–NW structure (i) and pseudocolor images for the illumination of linearly polarized laser light (ii). The linearly polarized laser light was directionally coupled toward the LO (red box) of the NW. **e** Normalized intensities of the LO and RO in the (L-handed chiral Au NC)@SiO_2–NW structures under the excitation of LCP/RCP light. The data were collected and averaged from 3 L-handed structures. The dashed arrows in (**e**) represent the range of measured normalized intensities. **f**, **g** Normalized intensities of the LO and RO in the (L-/D-handed chiral Au NC)@SiO_2–NW structures (**f**) and directionality of the surface plasmon polariton propagation (**g**) under the excitation of linearly polarized light. The data were collected and averaged from 3 L-/D-handed structures. The dashed arrows in (**f**, **g**) represent the range of measured normalized intensities.

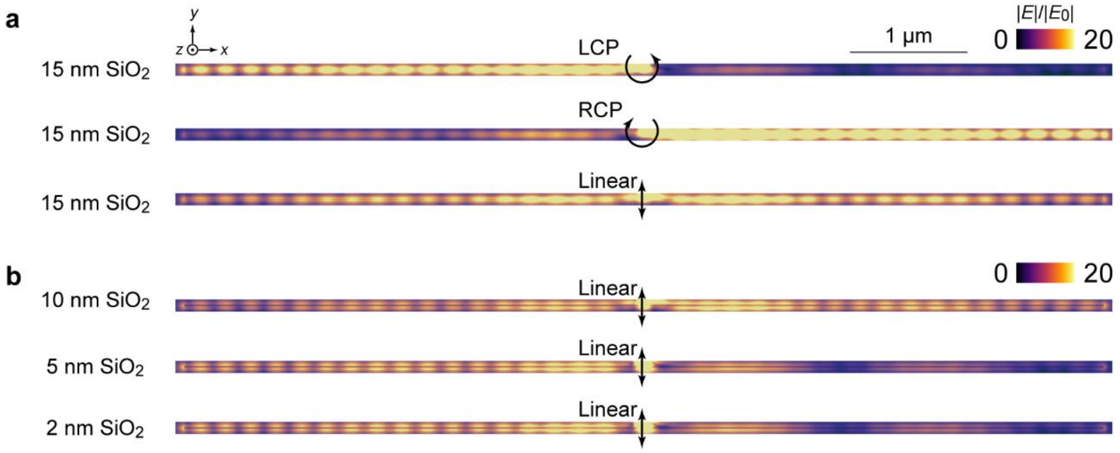

Supplementary Fig. 24 | Simulations of the electric field of the (L-handed chiral Au nanocube)@silica–(Ag nanowire) structures with different shell thicknesses. a Distributions of the electric field enhancement in an *x*-*y* plane monitor located at the bottom surface of the Ag nanowire (NW) in the (L-handed chiral Au nanocube)@15-nm-SiO_2–(Ag NW) structure under the excitation of circularly and linearly polarized dipole sources. Surface plasmon polaritons (SPPs) propagate along the –*x* direction for the left-handed circularly polarized dipole, whereas SPPs propagate along the +*x* direction for the right-handed circularly polarized dipole. **b** Distributions of the electric field enhancement for the (L-handed chiral Au nanocube)@SiO_2–(Ag NW) structures with different shell thicknesses under the excitation of linearly polarized dipole sources. The SiO_2 thicknesses are 10 nm, 5 nm, and 2 nm, respectively. SPPs propagate preferentially along the +*x* direction when the shell thickness is 10 nm or 15 nm. Reducing the SiO_2 thickness from 10 nm to 5 nm results in a reversal of the SPP propagation direction, from +*x* to –*x*.

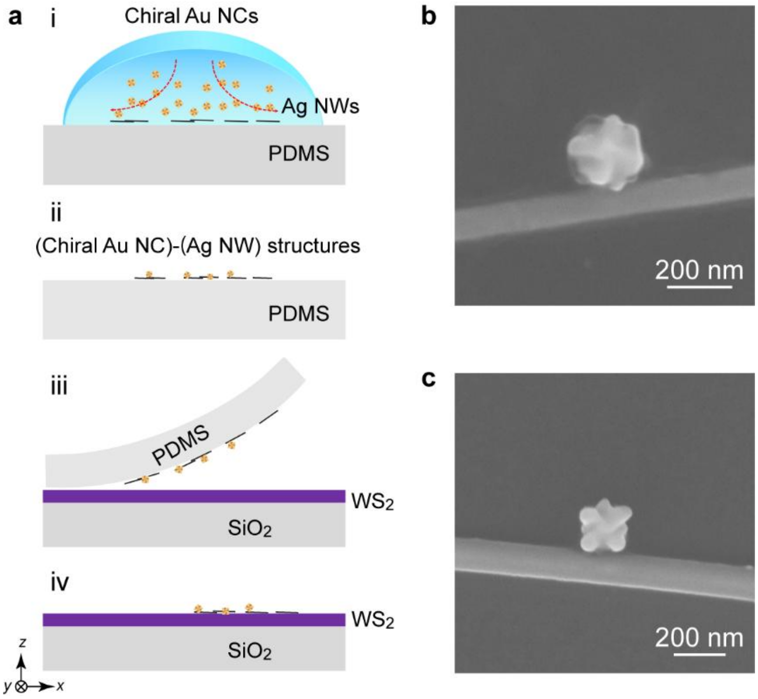

Supplementary Fig. 25 | Fabrication of the (chiral Au nanocube)–(Ag nanowire)-on-WS_2 structures. a Schematic illustrating the transfer of the chiral Au nanocube–nanowire (NC–NW) structures from a polydimethylsiloxane (PDMS) substrate to a WS_2 monolayer. **b** Scanning electron microscopy (SEM) image of a (D-handed chiral Au NC)–(Ag NW)-on-WS_2 structure. **c** SEM image of an (L-handed chiral Au NC)–(Ag NW)-on-WS_2 structure.

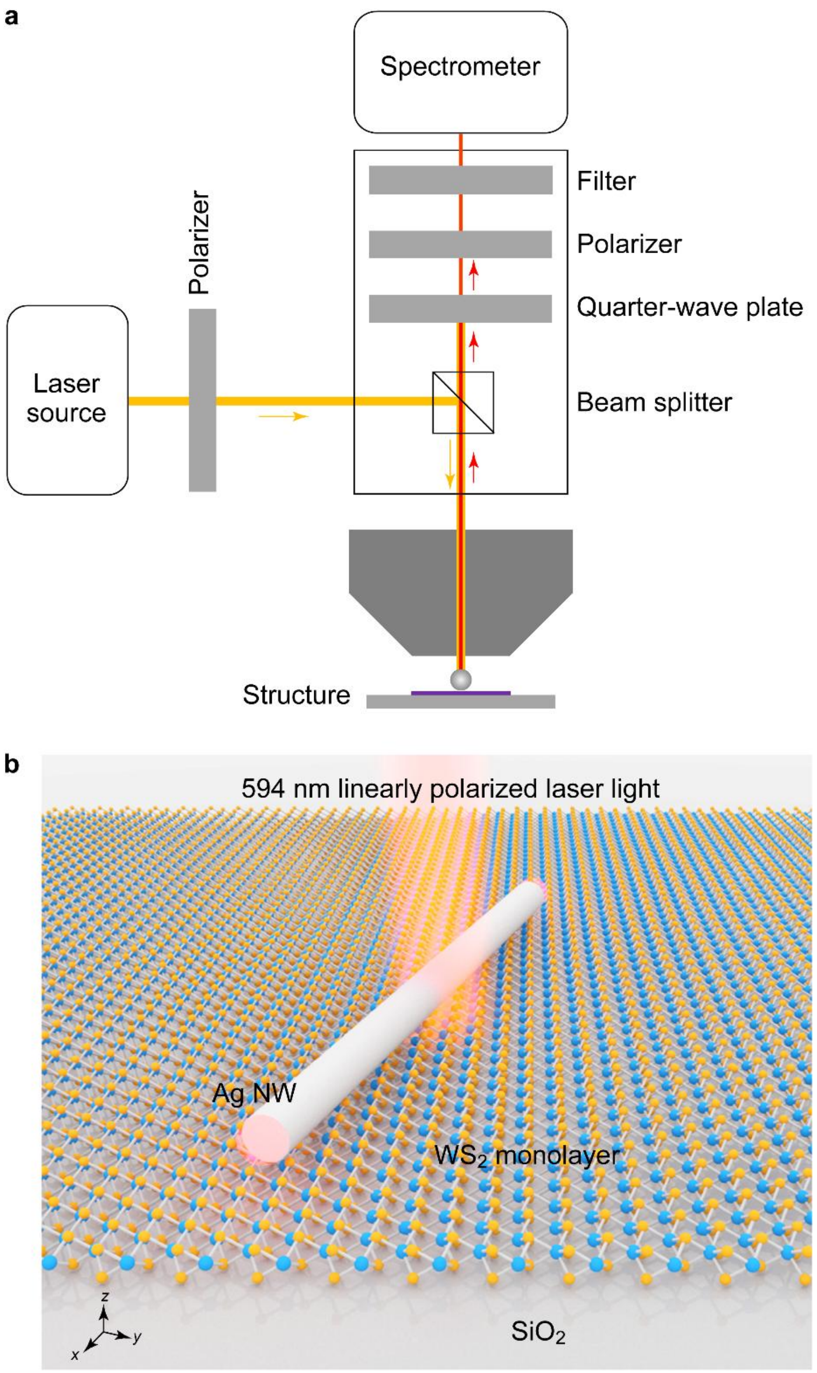

Supplementary Fig. 26 | Schematics of the experimental setup and (Ag nanowire)-on-WS_2 structure. **a** Experimental setup of polarization-resolved photoluminescence (PL) spectroscopy. **b** (Ag nanowire)-on-WS_2 structure. The laser was focused on the central *y*-axis of the Ag nanowire and ~0.3 μm away from the central *x*-axis of the Ag nanowire.

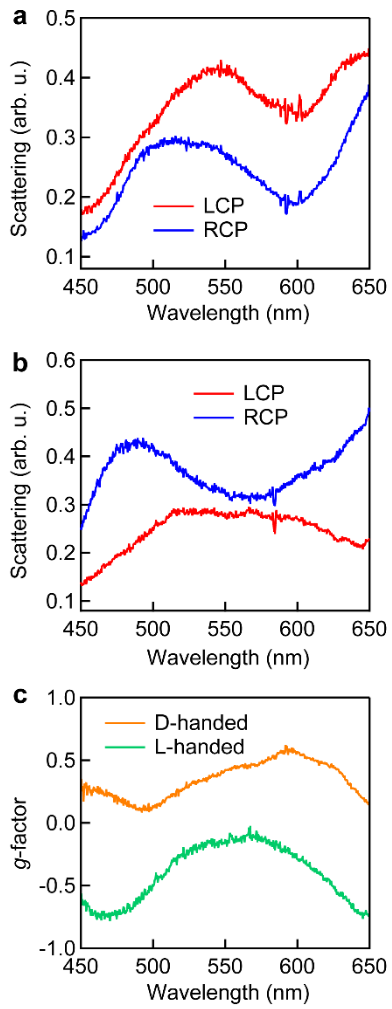

Supplementary Fig. 27 | Circular differential scattering measurements of the chiral Au nanocubes in the (chiral Au nanocube)–(Ag nanowire)-on-WS_2 structures. a, **b** Scattering spectra of the D-handed (**a**) and L-handed chiral Au nanocubes (NCs) (**b**) in the hybrid structures under circularly polarized light excitation. **c** Scattering *g*-factor spectra of the D-handed and L-handed chiral Au NCs in the hybrid structures.

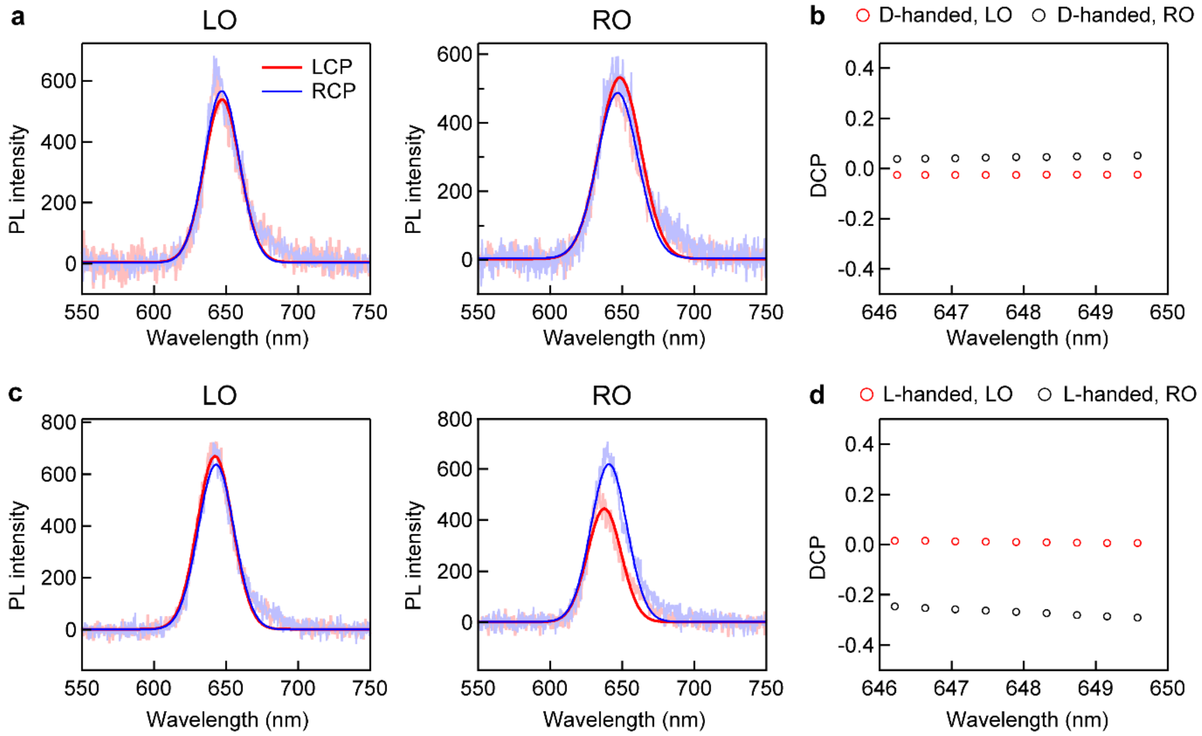

Supplementary Fig. 28 | Polarization-resolved photoluminescence spectra and degrees of circular polarization of the photoluminescence signals. a, **b** Polarization-resolved spectra (**a**) and degrees of circular polarization (DCPs) (**b**) of the photoluminescence (PL) at the right and left output (RO and LO) in the (D-handed chiral Au nanocube)–(Ag nanowire)-on-WS_2 structure under the excitation of 514 nm linearly polarized laser light. **c**, **d** Polarization-resolved spectra (**c**) and DCPs (**d**) of the PL at the RO and LO in the (L-handed chiral Au nanocube)–(Ag nanowire)-on-WS_2 structure under the excitation of 514 nm linearly polarized laser light.

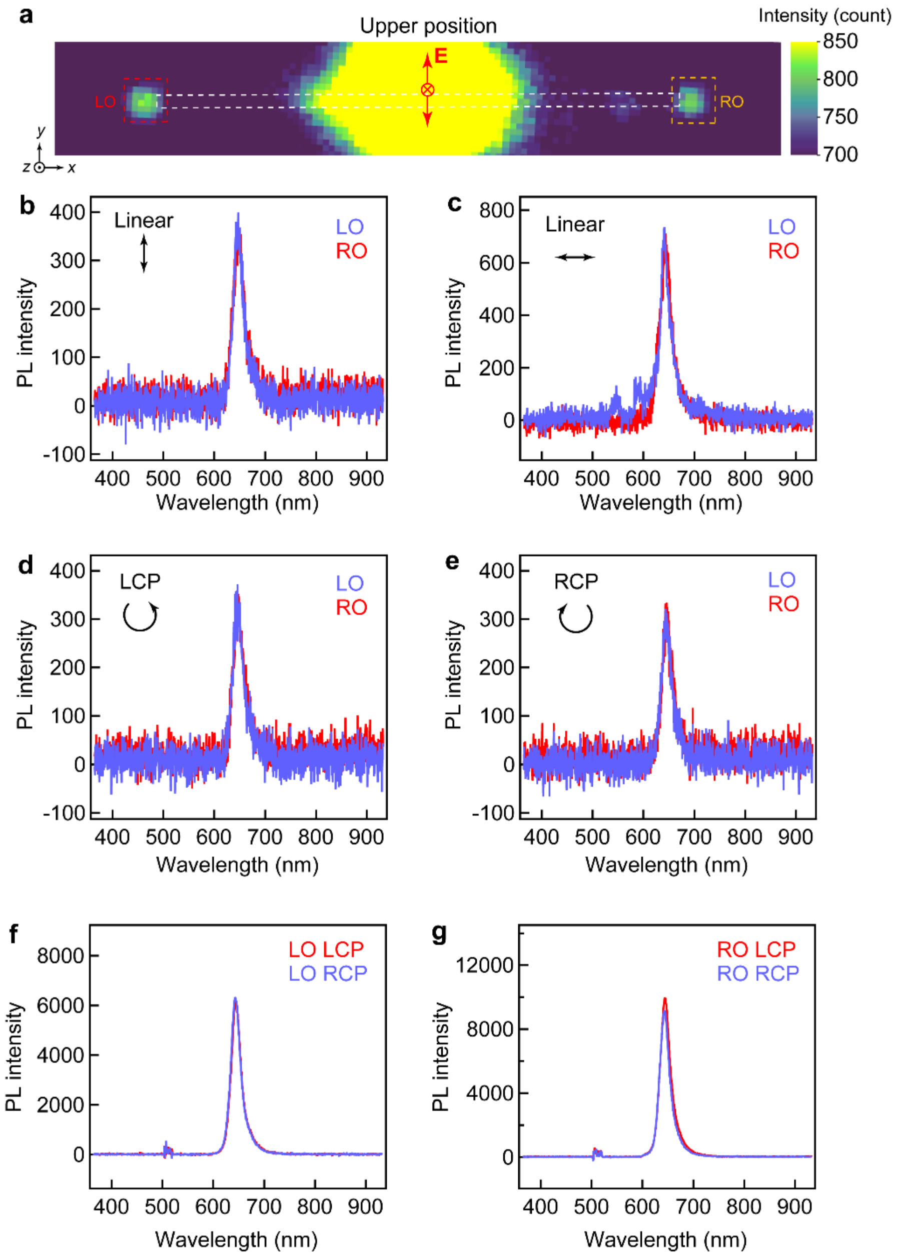

Supplementary Fig. 29 | Photoluminescence spectra and polarization-resolved photoluminescence spectra of the outputs in the (Ag nanowire)-on-(WS_2 monolayer) structure under 514 nm laser excitation. a Pseudocolor image of the structures for the illumination of linearly polarized laser light. The laser was focused on the upper side of nanowire (NW) with a lateral offset of ~150 nm from its center. **b**, **c** Photoluminescence (PL) spectra collected from the left and right output (LO and RO) under linear polarization excitation, with polarization perpendicular (**b**) and parallel (**c**) to the long axis of the Ag NW. **d**, **e** PL

spectra of the LO and RO for the illumination of left- (**d**) and right-handed circularly polarized (LCP and RCP) laser light (**e**). **f**, **g** Polarization-resolved PL spectra of LO (**f**) and RO (**g**) for the illumination of linearly polarized laser light. The laser power in (**f**, **g**) was increased 30-fold to improve signal-to-noise ratio. The PL outputs from the LO and RO of the NW remained symmetric under linear or circular polarization excitation. The difference between the LCP and RCP components of LO/RO was minimal.

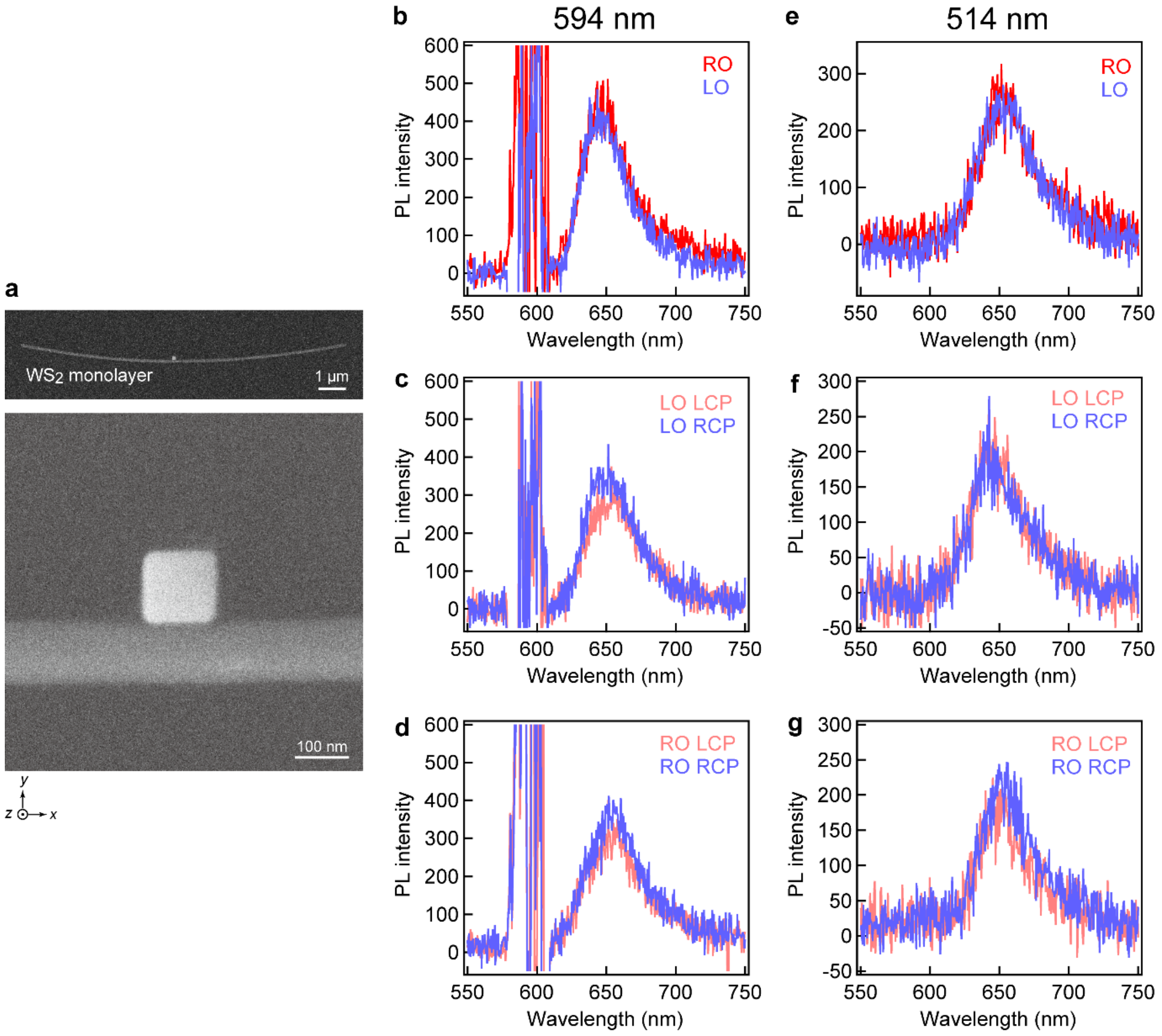

Supplementary Fig. 30 | Photoluminescence spectra and polarization-resolved photoluminescence spectra of the outputs of the (Au nanocube)–(Ag nanowire)-on-WS_2 structure. a Scanning electron microscopy images of a (Au nanocube)–(Ag nanowire)-on-WS_2 structure. **b** Photoluminescence (PL) spectra of the left and right output (LO and RO) under the excitation of 594 nm linearly polarized laser light. **c**, **d** Polarization-resolved PL spectra of the LO (**c**) and RO (**d**) under the excitation of 594 nm linearly polarized laser light. **e** PL spectra of the LO and RO under the excitation of 514 nm linearly polarized laser light. **f**, **g** Polarization-resolved PL spectra of the LO (**c**) and RO (**d**) under the excitation of 514 nm linearly polarized laser light.

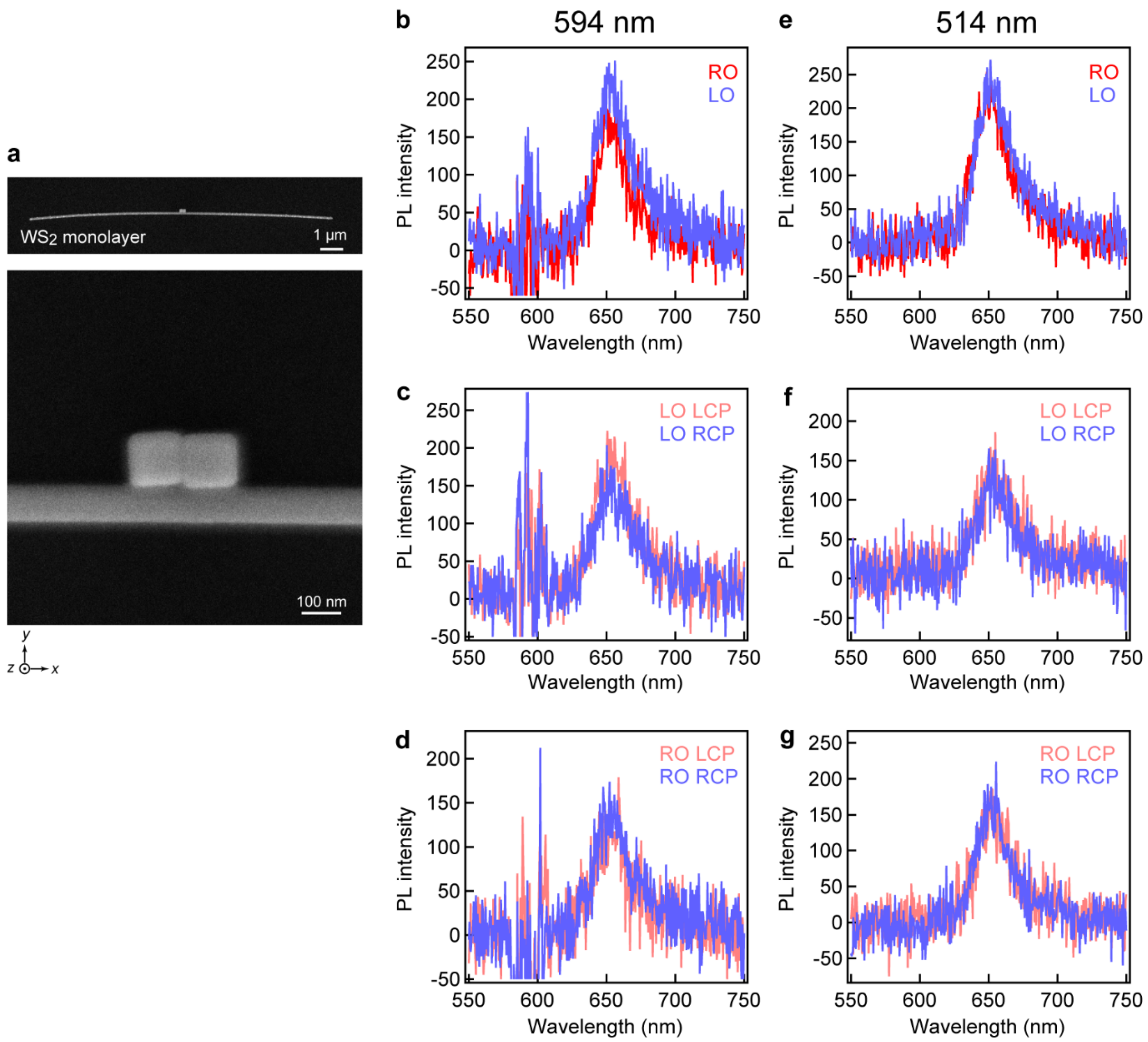

Supplementary Fig. 31 | Photoluminescence spectra and polarization-resolved photoluminescence spectra of the outputs of the (Au nanocube dimer)–(Ag nanowire)-on-WS_2 structure. a Scanning electron microscopy images of a (Au nanocube dimer)–(Ag nanowire)-on-WS_2 structure. **b** Photoluminescence (PL) spectra of the left and right output (LO and RO) under the excitation of 594 nm linearly polarized laser light. **c**, **d** Polarization-resolved PL spectra of the LO (**c**) and RO (**d**) under the excitation of 594 nm linearly polarized laser light. **e** PL spectra of the LO and RO under the excitation of 514 nm linearly polarized laser light. **f**, **g** Polarization-resolved PL spectra of the LO (**c**) and RO (**d**) under the excitation of 514 nm linearly polarized laser light.

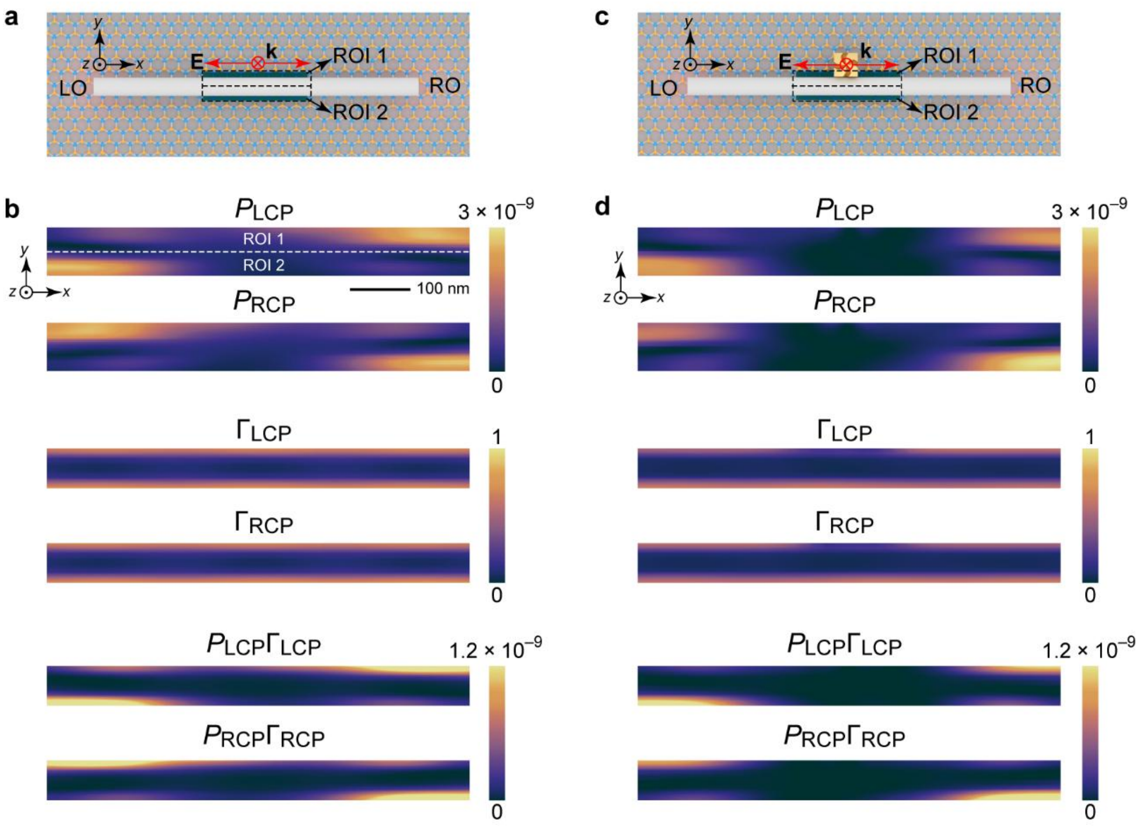

Supplementary Fig. 32 | Calculations of the emission efficiencies of valley excitons under the excitation of a 594 nm linearly polarized Gaussian beam. a Schematic of the (Ag nanowire)-on-WS_2 structure. **b** Distributions of the P_{LCP}, P_{RCP}, Γ_{LCP}, Γ_{RCP}, $P_{LCP}\Gamma_{LCP}$, and $P_{RCP}\Gamma_{RCP}$ of the (Ag nanowire)-on-WS_2 structure. Subscript LCP/RCP represents left-/right-handed circularly polarized. **c** Schematic of the (D-handed chiral Au nanocube)–(Ag nanowire)-on-WS_2 structure. **d** Distributions of the P_{LCP}, P_{RCP}, Γ_{LCP}, Γ_{RCP}, $P_{LCP}\Gamma_{LCP}$, and $P_{RCP}\Gamma_{RCP}$ of the (D-handed chiral Au nanocube)–(Ag nanowire)-on-WS_2 structure. The Gaussian beam is incident on the same position for the simulations of the (Ag nanowire)-on-WS_2 structure and (chiral Au nanocube)–(Ag nanowire)-on-WS_2 structure. All the images in (**b**, **d**) have the same scale bar.

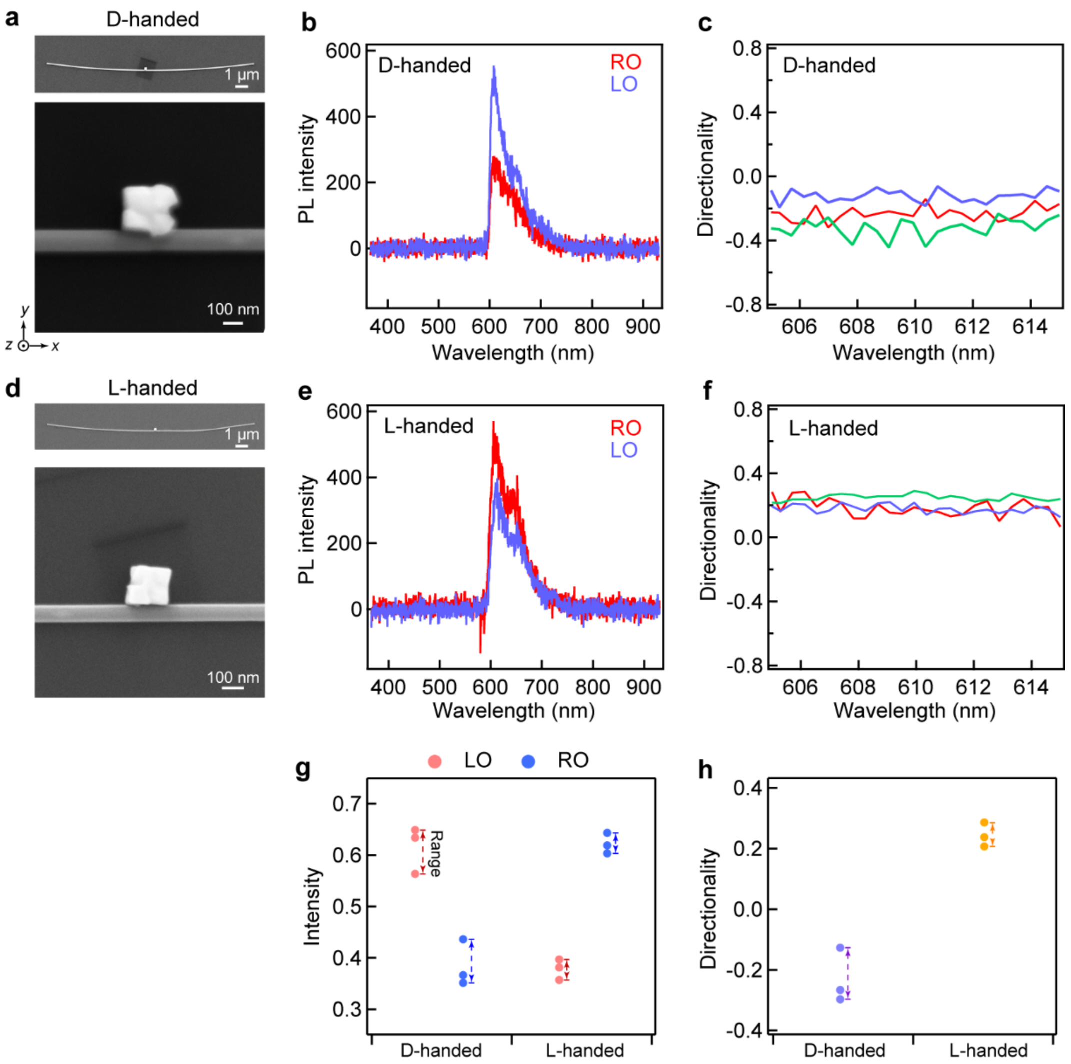

Supplementary Fig. 33 | Directional photoluminescence from the R640-coupled (chiral Au nanocube)–nanowire structures under the excitation of linearly polarized laser light. **a**, **b** Scanning electron microscopy (SEM) images of a (D-handed chiral Au nanocube)@R640–(Ag nanowire) structure (**a**) and photoluminescence (PL) spectra of the left and right output (LO and RO) (**b**). The direction of linear polarization of the laser light is perpendicular to the long axis of the Ag nanowire. The fluorophore molecules were embedded in a mesostructured SiO_2 shell surrounding the chiral Au nanocube. **c** Directionality of the surface plasmon polariton (SPP) propagation of 3 (D-handed chiral Au nanocube)@R640–(Ag nanowire) structures. **d**, **e** SEM images of a (L-handed chiral Au nanocube)@R640–(Ag nanowire)

structure (**d**) and PL spectra of the LO and RO (**e**). **f** Directionality of the SPP propagation of 3 (L-handed chiral Au nanocube)@R640–(Ag nanowire) structures. **g**, **h** Normalized intensities of the LO and RO in the (D-/L-handed chiral Au nanocube)–nanowire structures (**g**) and averaged directionality of the PL SPP propagation (**h**). The data were collected and averaged from 3 (L-/D-handed chiral Au nanocube)–nanowire structures at the wavelength of 606 nm. The dashed arrows in (**g**, **h**) represent the range of measured normalized intensities.

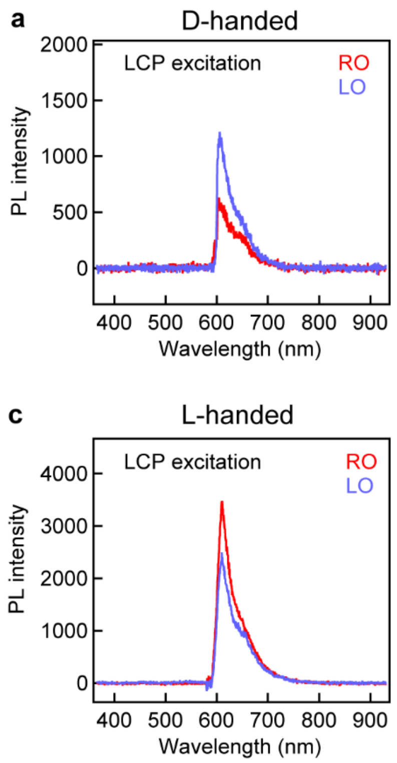

Supplementary Fig. 34 | Photoluminescence spectra of the outputs in the (D-/L-handed chiral Au nanocube)@R640–nanowire structures under the excitation of circularly polarized laser light. a, **b** Photoluminescence (PL) spectra of the left and right output (LO and RO) of a (D-handed chiral Au nanocube)@R640–(Ag nanowire) structure for the illumination of left- (**a**) and right-handed circularly polarized (LCP and RCP) laser light (**b**). The D-handed structure exhibits PL propagation toward the LO. The difference between the LO and RO under LCP excitation is larger than that under RCP excitation. **c**, **d** PL spectra of the LO and RO of a (L-handed chiral Au nanocube)@R640–(Ag nanowire) structure for the illumination of LCP (**c**) and RCP (**d**) laser light. The L-handed structure exhibits PL propagation toward the RO. The difference between the LO and RO under RCP excitation is larger than that under LCP excitation.

Supplementary Note 1

Surface plasmon polariton (SPP) modes are supported by Ag nanowires (NWs) embedded in a surrounding dielectric environment. The solutions to Maxwell's equations for the electromagnetic field were utilized to calculate the fundamental modes of an infinitely long Ag NW. The NW with the electric permittivity of ε_2 was modeled as a cylinder with a certain radius R. The NW was positioned along the x axis and surrounded by a dielectric medium with the electric permittivity of ε_1. For a nonmagnetic medium, the electric and magnetic fields satisfy the wave equations of

$$\nabla^2\mathbf{E}(\mathbf{r}) + \frac{\omega^2}{c^2}\varepsilon(\mathbf{r})\mathbf{E}(\mathbf{r}) = 0 \tag{S1}$$

$$\nabla^2\mathbf{H}(\mathbf{r}) + \frac{\omega^2}{c^2}\varepsilon(\mathbf{r})\mathbf{H}(\mathbf{r}) = 0 \tag{S2}$$

The solutions to Eqs. S1 and S2 are the distributions of the electric and magnetic fields ($\mathbf{E}_i$ and $\mathbf{H}_i$) for the plasmon modes of the cylindrical NW, where i = 1, 2 denote the regions outside and inside the cylinder, respectively. The electric field distribution at the coordinates of (x, y, z) can be written as $\mathbf{E}_i(x, y, z) = \mathbf{E}_i(\mathbf{r})e^{im\varphi+ik_\parallel x}$. $\mathbf{E}(\mathbf{r})$ is the radial distribution of the electric field, where $\mathbf{r}$ represents the distance from the center of the NW ($r^2 = y^2 + z^2$). φ is the azimuthal angle in the cylindrical coordinate system. m is an integer characterizing the winding of the mode. $k_\parallel$ is the longitudinal component of the wavevector along the length axis of the Ag NW, which is related to the vacuum wavevector $k_0 = \omega/c$, electric permittivity ε_i, and the transverse component of the wavevector $k_{i\perp}$ by the equation of $\varepsilon_i k_0^2 = k_\parallel^2 + k_{i\perp}^2$. The similar expression of the magnetic field $\mathbf{H}_i(x, y, z)$ can also be obtained. The coefficients in the expressions of $\mathbf{E}_i(x, y, z)$ and $\mathbf{H}_i(x, y, z)$ were further determined by enforcing the boundary conditions on the tangential components E_φ, E_x, H_φ, and H_x of the fields to be continuous at the metal–dielectric interface. A nontrivial solution for the mode equations of $\mathbf{E}_i$ and $\mathbf{H}_i$ was obtained. The mode equations determine the allowed values of $k_\parallel$ as a function of m, R, and ε_i.

For circularly polarized light (CPL) propagating in the $-y$ direction, LCP and RCP light can be expressed as $\sigma_{\mathrm{LCP}} = (-\mathbf{e}_x + i\mathbf{e}_z) / \sqrt{2}$ and $\sigma_{\mathrm{RCP}} = (-\mathbf{e}_x - i\mathbf{e}_z) / \sqrt{2}$, respectively. The coupling efficiency between CPL and the electric field distributions $|\mathbf{E}(y, z)|$ of the plasmon modes can be evaluated by the overlapping intensities $S_{\mathrm{LCP}} = |\sigma_{\mathrm{LCP}}{}^{*}\cdot\mathbf{E}(y, z)|^2$ and $S_{\mathrm{RCP}} = |\sigma_{\mathrm{RCP}}{}^{*}\cdot\mathbf{E}(y, z)|^2$. The calculated results show that the S_{LCP} and S_{RCP} values are localized on the surface of the NW (Supplementary Fig. 14). The electric field distributions $|\mathbf{E}(y, z)|$ of the TM_0, HE_{+1}, and HE_{-1} modes were obtained as shown in Supplementary Fig. 13. The superscripts $+x$ of the TM and HE modes represent the propagation direction of the SPP modes. The distribution of the overlapping intensity between CPL and the $\mathrm{HE}_1{}^{+x}$ mode was reversed when the polarization of the incident light was switched from LCP to RCP (Supplementary Fig. 14c, d). Such a mirror symmetry of S_{LCP} and S_{RCP} is also valid for the distributions of the overlapping intensities for CPL and the $\mathrm{HE}_{-1}{}^{+x}$ mode (Supplementary Fig. 14e, f).

Supplementary Note 2

The overlapping intensities between CPL propagating along the −*x*, −*y*, or −*z* directions and the FDTD-calculated distribution of $|\mathbf{E}(y, z)|/|E_0|$ of the HE modes are displayed in Supplementary Fig. 18. When a LCP light incident along the −*y* direction is coupled to the lower surface of the NW, the coupled SPPs will propagate along the NW in the +*x* direction (Supplementary Fig. 18a, b). Conversely, an incident RCP light should be coupled to the upper surface of the NW to achieve SPP propagation along the +*x* direction. For LCP and RCP light incident in the −*z* direction, the overlapping intensities of the HE mode propagating in the +*x* direction are distributed on the right and left half surfaces of the NW, respectively. The distribution of the overlapping intensities between CPL with −*y* or −*z* propagating directions and the HE modes are reversed when the propagating direction of the hybrid mode is changed from +*x* (Supplementary Fig. 18a, b) to −*x* (Supplementary Fig. 18c, d), indicating the presence of the spin-Hall effect. However, for incident CPL propagating along the −*x* direction, the distributions of the overlapping intensities show that the photonic spin-Hall effect is invalid.

When an incident CPL propagating along the −*x* or −*y* direction is uniformly coupled at the +*y* side of the NW surface, the photonic spin-Hall effect is invalid. The reason for the invalidity is that the $S_{\mathrm{LCP}}/|E_0|^2$ value of the HE^{+x} mode (Supplementary Fig. 18a, b) and the $S_{\mathrm{LCP}}/|E_0|^2$ value of the HE^{-x} mode (Supplementary Fig. 18c, d) distributed on the +*y* side of the NW surface show equal overall overlapping intensities. Akin to $S_{\mathrm{LCP}}/|E_0|^2$, the $S_{\mathrm{RCP}}/|E_0|^2$ value of the HE^{+x} mode and the $S_{\mathrm{RCP}}/|E_0|^2$ value of the HE^{-x} mode distributed on the +*y* side of the NW surface show equal overall overlapping intensities. We therefore attribute the photonic spin-Hall effect primarily to the near-field coupling between the *z*-component of the electric field and the plasmon modes of the Ag NW.
